# mTACT: A cell type-specific transportome-scale amiRNA toolbox to overcome functional redundancy in Arabidopsis

**DOI:** 10.1093/plphys/kiaf682

**Published:** 2025-12-31

**Authors:** Moran Anfang, Shir Ben Yaakov, Ning Su, Anat Shafir, Jenia Binenbaum, Reem Haj Yahya, Xikai Yu, Carl Procko, Hamtual Bar, Joanne Chory, Julian I. Schroeder, Yosef Fichman, Itay Mayrose, Eilon Shani, Yuqin Zhang

**Affiliations:** 1 School of Plant Sciences and Food Security, Tel Aviv University, Tel Aviv, 69978, Israel; 2 College of Advanced Agricultural Sciences, University of Chinese Academy of Sciences, Beijing, 100101, China; 3 Institute of Genetics and Developmental Biology, Chinese Academy of Sciences, Beijing, 100101, China; 4 Plant Biology Laboratory, The Salk Institute for Biological Studies, La Jolla, California, 92037, USA; 5 Cell and Developmental Biology Department, School of Biological Sciences, University of California San Diego, La Jolla, CA, 92093-0116 USA

## Abstract

In plants, both developmental processes and environmental responses are spatiotemporally regulated by an assembly of signaling molecules such as hormones, secondary metabolites, and ions. The ability of these signaling molecules to move within and across plant tissues is essential for various developmental cues. However, the characterization of transported signaling molecules and their translocation mechanisms is difficult due to the functional redundancy of plant genomes and shortcomings in methodologies. Here, we report our development of the Multi Targeted AmiRNA Cell type-specific Transportome-scale (mTACT) toolbox, which can be used to reveal phenotypic plasticity in plants. mTACT is based on a large set of artificial microRNAs (amiRNAs), each designed to optimally target multiple members of a particular gene family encoding transporter proteins. In total, the mTACT toolbox includes 5,565 amiRNAs, targeting 81.7% of the *Arabidopsis* (*Arabidopsis thaliana*) transportome. The amiRNA library can be driven under 12 cell type-specific promoters, allowing the design of spatial-specific genetic screens. mTACT is further divided into eight sub-libraries of amiRNAs targeting a functionally defined protein class. A proof-of-concept screen validated the mTACT approach by identifying phenotypes linked to both known and unidentified genes. With the ability to overcome functional redundancy in a transportome-scale, cell type-specific manner, the mTACT toolbox will allow the plant research community to study previously hidden genetic factors required for long- and short-distance translocation of signaling molecules.

## Introduction

Plant hormones, secondary metabolites, ions, small peptides, and other small molecules govern key developmental processes in plants and their response to the environment (Davies, 2010). The activity of signaling molecules is controlled at multiple levels of metabolism, distribution, perception, and transduction. Modulation of these regulatory steps directly impacts downstream responses such as target gene expression and protein activity (Grienenberger and Fletcher, 2015). In addition, signaling molecules are often produced in only certain cell types and may act locally or in distant cells and tissues (Santner et al., 2009; Lacombe and Achard, 2016; Ruffel, 2018; Hirayama and Mochida, 2022). Non-cell-autonomous processes require that molecules, or other signals, move in and out of cells by short distances, symplasticly or apoplasticly, among cells within a particular tissue, or over long distances through the vascular system (Khakhar et al., 2018). Monitoring the movement and distribution of these signal molecules in a quantitative and non-invasive manner is essential for a thorough understanding of their regulatory functions (Santner et al., 2009; Khakhar et al., 2018). However, imaging the movement of small molecules at high resolution remains challenging, making it difficult to quantify the spatial distribution of signaling molecules and hindering genetic screens to identify transporters involved in signaling molecule movement (Abualia et al., 2018; Anfang and Shani, 2021).

For many decades, forward genetics has been a powerful approach to the identification of genes and mutations that underlie phenotypes of interest in plants (Khalil et al., 2018), including those that encode transporters. However, genetic analysis of transporter gene families has been precluded in many cases due to the lack of observable phenotypes in single-gene, loss-of-function mutations. Partial, complete, or conditional functional redundancy between different family members results in the absence of visible phenotypes in single-gene knockouts. Therefore, high-order mutants of redundant genes from the same gene family are needed to reveal or increase the severity of phenotypes (Bouché and Bouchez, 2001; Lacombe and Achard, 2016; Park et al., 2017).

Ancient duplication events and a high retention rate of genes with similar amino acid sequences have contributed to the abundance of duplicated genes in plant genomes. For example, an average of 65% of plant genes are paralogous, ranging from 46% in the moss *Physcomitrium patens* to 84% in the apple *Malus domestica* (Panchy et al., 2016). Of the approximately 25,000 genes in *Arabidopsis*, 75% belong to gene families with at least two members (Bouché and Bouchez, 2001; Kafri et al., 2009; Hauser et al., 2013; Panchy et al., 2016). This robust genetic redundancy allows plant adaptation to the ever-changing environment (Hwang et al., 2016; Panchy et al., 2016). Genetic redundancy is tightly connected to mechanistic cellular networks (Bouché and Bouchez, 2001; Kafri et al., 2009; Li et al., 2015; Wendrich et al., 2020). For example, the NPF transporter family contains 53 genes and the ABC transporter family contains 130 genes in *Arabidopsis* (Sánchez-Fernández et al., 2001; Cheng et al., 2022). These genes exhibit redundant phenotypes despite the somewhat diverse substrate specificities of the encoded proteins (Hwang et al., 2016; Binenbaum et al., 2018; Do et al., 2018). In order to identify novel activities of redundant genes, overexpression strategies have often been used. However, such approaches can lead to pleiotropic phenotypes that may not be relevant to the biological activity of the studied protein (Weigel et al., 2000). Therefore, it is of high priority to generate visible loss-of-function mutations in a forward-genetics manner. Significant progress has been made in recent years using genome-scale RNA interference methods and artificial microRNAs (amiRNAs) (Schwab et al., 2006; Ossowski et al., 2008). The amiRNAs are generated using an endogenous miRNA backbone as a template, replacing the target recognition sequence with a custom sequence. amiRNA precursors can be computationally designed to target a specific group of potentially redundant genes; in the plant, the amiRNA precursor is specifically processed to yield mature amiRNA (Alvarez et al., 2006; Schwab et al., 2006). As a tool to overcome the obstacle of functional redundancy, a computationally designed list of over 2 million amiRNAs, termed Phantom Database, was generated (Hauser et al., 2013). From these computationally designed amiRNAs, 22,000 amiRNAs were synthesized for library generation at combinatorial targeting of 18,117 genes. 96% of the synthesized amiRNAs in the library were predicted to target two to five potentially redundant genes identified by shared sequence homology (Hauser et al., 2013). The Phantom library was divided into 10 sub-libraries; each containing 1,505-4,082 amiRNAs targeting a functionally defined protein class. By overcoming functional redundancy and bypassing the genetic linkage of multiple knockdowns, the Phantom library, driven by a constitutive *35S* promoter, allows rapid mapping of newly recognized homologous candidate mutant genes without the need for laborious map-based cloning. The use of this library resulted in the identification of dozens of redundant phenotypes (Hauser et al., 2019; Takahashi et al., 2020; Xie et al., 2021), including several previously unknown hormone transporters (Zhang et al., 2018; Zhang et al., 2021; Chen et al., 2023).

Notably, the Phantom amiRNAs are driven by the strong constitutive *35S* promoter. This has several drawbacks: first, expression from the *35S* promoter may result in a decay of amiRNA-mediated silencing over generations. Additionally, in rare cases, it can result in lethal phenotypes and pleiotropic effects across different cell or tissue types. Second, only 1,777 amiRNAs in the Phantom library target transporter genes (Zhang et al., 2018). Third, its algorithm design allowed amiRNA to target genes from the same family but did not account for the phylogenetic relationships among family members. Consequently, in some cases, the designed amiRNAs targeted only distantly related genes, reducing the probability of targeting genes with overlapping functions. A recent approach used CRISPR libraries to multi-target several genes from the same family (Hu et al., 2023; Lorenzo et al., 2023). This included a genome-scale library in *Arabidopsis* (Hu et al., 2023), and tomato (Berman et al., 2025). However, these tools were constructed using a ubiquitous or meristematic promoter that generates inherited mutations in all plant cells with no cell-type or tissue resolution.

In order to address these limitations and to allow the identification of redundant transportome components, we have developed the Multi Targeted AmiRNA Cell type-specific Transportome-scale (mTACT) toolbox, a system for knockdown gene families at the transportome scale and the cell type level. The mTACT library contains 5,565 amiRNAs, each designed to target two to eight closely homologous genes within transporter gene families in a variety of combinations (Fig. S1). The genetic toolbox developed here will allow scientists to overcome functional redundancy in a forward-genetic, targeting specific transporter genes from defined families in a tissue-specific manner.

## Results

### Constructing cell type and tissue-specific vectors to drive multi-targeted amiRNAs

Conventional forward genetic screens often struggle to detect homologous genes with redundant functions. To reveal loss-of-function phenotypes in such cases, simultaneous silencing of multiple gene family members is necessary. The Phantom library, developed to target homologous genes in *Arabidopsis* (Hauser et al., 2013; Zhang et al., 2018), has certain limitations, including the progressive reduction of amiRNA-mediated silencing over generations, potential lethality, and unintended pleiotropic effects that may complicate phenotype analysis. To address these challenges, we aimed to construct a library of amiRNAs to target multiple members of each transporter gene family across the *Arabidopsis* genome in a cell type-specific manner. We envisioned that the tool would allow the design of spatial forward-genetics screens, expanding our ability to identify signaling molecules produced in one cell type that act in a distal tissue, an approach not available to the plant genetic community thus far.

**Fig. 1:**
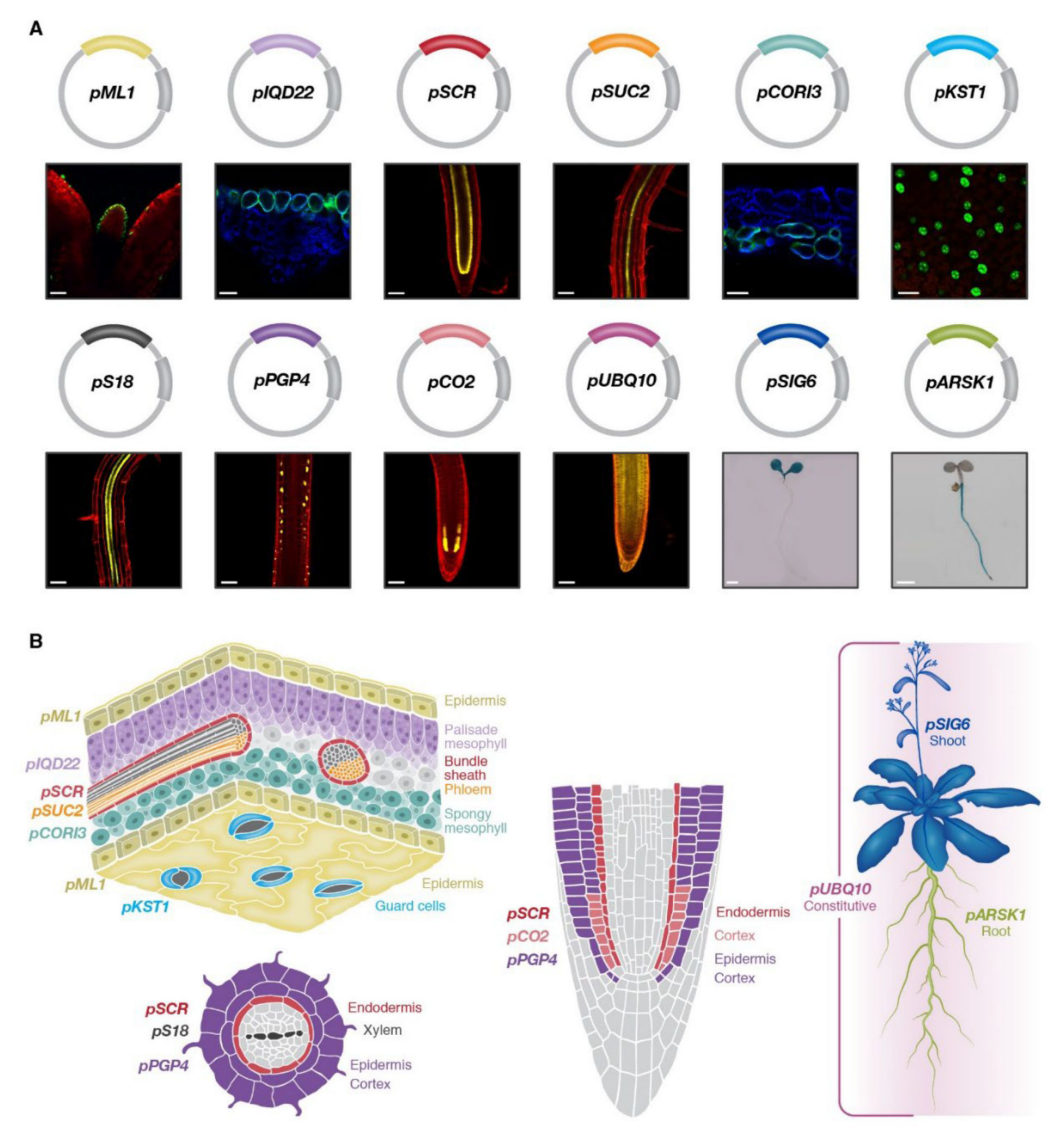
Expression pattern of cell type-specific promoters used to drive multi-targeted amiRNA libraries. **A)** Shown are confocal or stereoscope images for *pML1:H2B-GFP* (shoot epidermis), *pIQD22:GUS-mCitrine* (palisade mesophyll), *pSCR:YFP* (endodermis and bundle sheath), *pSUC2:YFP* (phloem companion cells), *pCORI3:GUS-mCitrine* (spongy mesophyll), *pKST1:GFP* (guard cells), *pS18:YFP* (xylem), *pPGP4:NLS-YFP* (root epidermis and cortex), *pCO2:YFP* (cortex), *pUBQ10:YFP* (constitutive), *pSIG6:GUS* (shoot), and *pARSK1:GUS* (root). Red signal indicates cell wall dye propidium iodide. Blue signal in *pIQD22* and *pCORI3* indicates chlorophyll auto-fluorescence. *pML1, pSCR, pSUC2, pKST1, pS18, pPGP4, pCO2*, and *pUBQ10* seedlings are 5 days old, *pSIG6* and *pARSK1* seedlings are 12 days old, and *pIQD22* and *pCORI3* are 17 days old. Scale bars = 50 µm for *pML1, pIQD22, pSCR, pSUC2, pCORI3, pKST1, pS18, pPGP4, pCO2*, and *pUBQ10*; scale bar = 1 mm for *pARSK1*; and scale bar = 2 mm for p*SIG6*. **B)** Illustrations of the cell type-specific expression patterns driven by promoters used in this study to drive multi-targeted amiRNA expression. Illustrations are of leaf tissue (upper left), root cross-section (lower left), root tip (center) and whole plant (right).

To allow library-level, cell type-specific amiRNA expression, we constructed 11 distinct cell type-specific promoter vectors: shoot epidermis (*pML1*) (Sessions et al., 1999), palisade mesophyll (*pIQD22*) (Procko et al., 2022), endodermis and bundle sheath (*pSCR*) (Malamy and Benfey, 1997), phloem companion cells (*pSUC2*) (Truernit and Sauer, 1995), spongy mesophyll (*pCORI3*) (Procko et al., 2022), guard cell (*pKST1*) (Kelly et al., 2017), xylem (*pS18*) (Marquès-Bueno et al., 2016), root-specific epidermis and cortex (*pPGP4*) (Cho et al., 2007), root cortex (*pCO2*) (Marquès-Bueno et al., 2016), shoot (*pSIG6*) (Hu et al., 2015), and root (*pARSK1*) (Hwang and Goodman, 1995), in addition to a constitutive promoter vector (*pUBQ10*) (Grefen et al., 2010) (Sup. Table 1). We confirmed the expression patterns of these promoters using NLS-YFP, YFP, H2B-GFP, GFP, or GUS reporters (Fig. 1). Outside of the leaf, *pCORI3* and *pIQD22* drove additional expression in root and stem cell types (Fig. S2, Fig. S3). All promoters were cloned into compatible vectors that allows for single-step highly efficient amiRNA cloning (Sup. Table 2). We expected that expression of the transportome-specific amiRNA libraries under different cell type-specific promoters would result in less silencing over generations and decreased probability of lethal and pleiotropic effects compared to the *35S* lines.

### Design of multi-targeted amiRNA transportome library in *Arabidopsis*

To knock down multiple homologous genes of the transportome simultaneously, we constructed the mTACT library of amiRNAs that are designed to target multiple members of each transporter gene family across the *Arabidopsis* transportome. The mTACT library has the following improvements relative to the Phantom transportome library: 1) an improved design that accounts for homologous relationships within gene families, thus increasing the probability that the designed amiRNAs will silence functionally redundant genes, 2) an expanded amiRNA arsenal of thousands of multi-targeted amiRNAs, and 3) targeted sub-libraries that allow exclusive amplification of amiRNAs targeting defined transporter gene families. We envisioned that mTACT would allow robust, high-quality, tissue-specific transportome analysis.

**Fig. 2:**
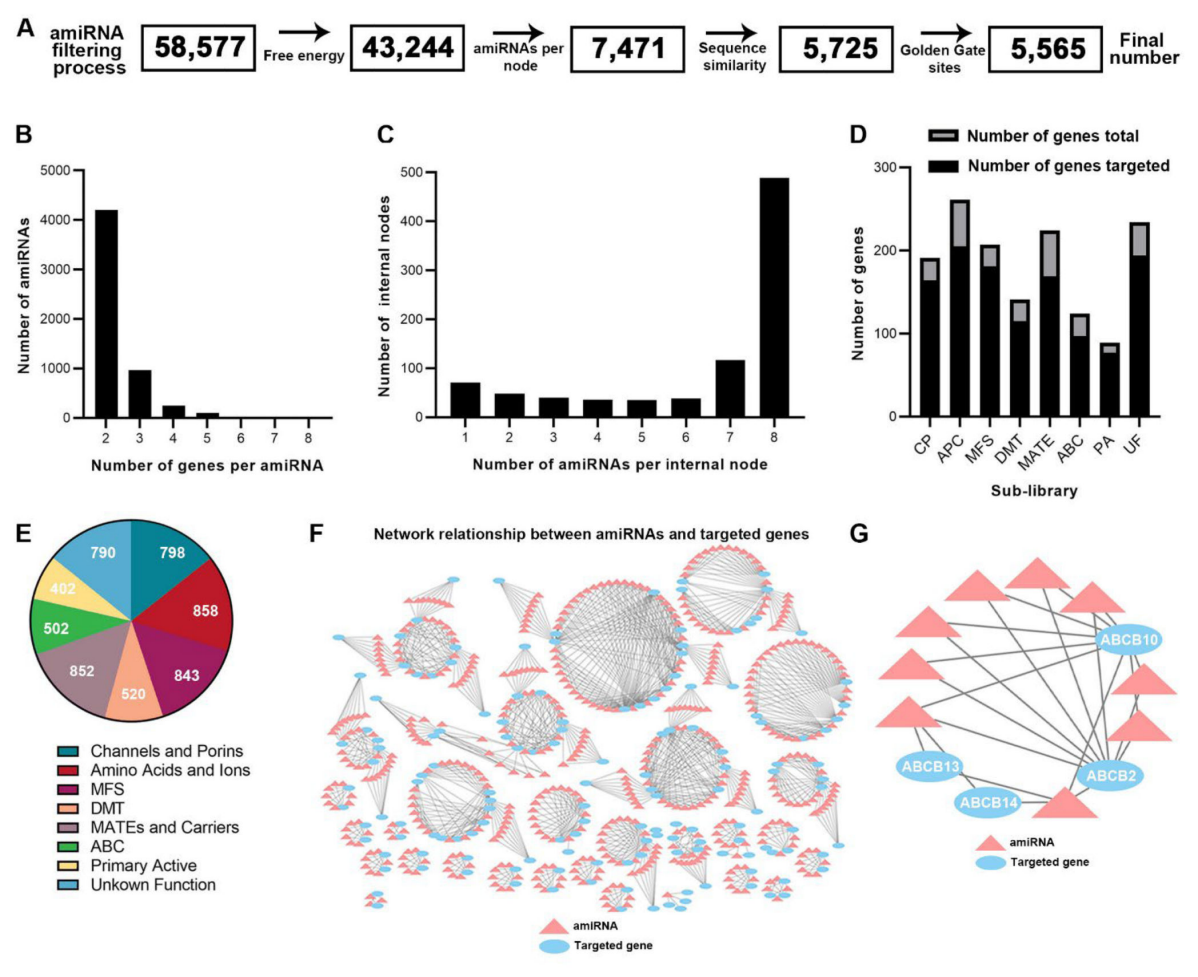
mTACT multi-targeted amiRNA library design. **A)** The amiRNA library was filtered based on free energy, number of amiRNAs per internal node, sequence similarity, and Golden Gate sites. **B)** Number of genes targeted per amiRNA for the entire mTACT library. **C)** Number of amiRNAs per internal node in the phylogenetic tree for the entire mTACT library. **D)** Number of genes targeted per total genes for each sub-library. CP, channels and porins; APC, amino acid/polyamine/organo-cation, cation carriers, and mitochondrial carriers; MFS, major facilitator superfamily; DMT, drug/metabolite transporter group; MATE, multi-drug and toxic compound extrusion and other carrier transporters; ABC, ATP binding cassette family; PA, primary active transporters; UF, unknown function. **E)** Number of amiRNAs in each sub-library. The total number of amiRNA is 5,565. **F)** Network relationship between amiRNAs (pink triangles) and target genes (blue ellipses) for the mTACT-ABC amiRNA library. **G)** Example of a typical mTACT network of ABCB family members (blue ellipse) targeted by amiRNAs (pink triangles). Black lines indicate for direct amiRNAs and target gene interactions.

To construct a library of amiRNAs that target multiple members of each gene family in the *Arabidopsis* transportome, we obtained a list of genes with putative transporter function, as annotated in the ARAMEMNON (Schwacke et al., 2003) and PLAZA (Proost et al., 2015) databases. After excluding singleton genes (i.e., genes with no family members) and genes without coding sequence data, the ARAMEMNON database includes 1,158 genes from 83 families, and the PLAZA database includes 1,325 genes from 189 families. Together the two classifications include 1,471 genes that encode putative transporters (Sup. Table 3). We then designed a set of amiRNAs that optimally target multiple members of each gene family while accounting for the similarity among family members (see Methods). Briefly, we first reconstructed the gene tree of each gene family. In these trees, homologous subgroups of genes that are closely related to each other are placed close together. We then traversed over the internal nodes of the tree and designed the optimal amiRNAs for each subgroup (i.e., the list of genes that are the descendants of the specified internal node) using the WMD3 program (Ossowski et al., 2008) and according to the hybridization energy of the amiRNAs, while excluding amiRNAs with likely off-target activity (Fig. 2A). The maximal number of amiRNAs per internal node was set to 8.

The mTACT library antisense and sense sequences were then introduced *in silico* into the *miRNA159a* backbone sequence (Palatnik et al., 2007). *amiRNA159a* has previously been shown to be non-mobile (Marín-González and Suárez-López, 2012), and efficiently silence gene expression in several studies in *Arabidopsis* (Rajagopalan et al., 2006; Furumizu et al., 2015). The entire amiRNA library was divided into eight different sub-groups using unique adaptors. These adaptors were added to allow amplification of a particular group of amiRNAs targeting a specific family or functional class (Sup. Tables 3 and 4). The groups include: channels and porins (CP); amino acid/polyamine/organo-cation, cation carriers, and mitochondrial carriers (APC); major facilitator superfamily (MFS); drug/metabolite transporter group (DMT); multi-drug and toxic compound extrusion and other carrier transporters (MATE); ATP binding cassette family (ABC); primary active transporters (PA); unknown function (UF). The resulting concatenated sequences (including backbone, antisense, sense, and adaptors) were removed if the sequence contained a Bsal restriction enzyme recognition site since Bsal was used for Golden Gate amiRNA library cloning.

Following the filtering procedure, the *in silico* library contained 5,565 amiRNA sequences, targeting 1,202 genes, 81.7% of the putative transporter-encoding genes in *Arabidopsis* (Sup. Table 3). Notably, 269 genes predicted to encode transporters (18.3 %) are not targeted by any amiRNA (Sup. Table 3). Each amiRNA targets two to eight genes from the same family (Fig. 2B, Fig. S4A, S5). Moreover, each internal node in the phylogenetic tree was targeted up to eight times (Fig. 2C, Fig. S4B, S6). Most of the amiRNAs target two genes, with a gradual decrease in the number of amiRNAs targeting three or more genes up to eight, which was set as a cut-off. Each gene is also targeted by more than one amiRNA, thus enabling various genetic combinations (Fig. S4-8). Furthermore, in each sub-group, the number of genes targeted is between 77 and 205 genes (Fig. 2D, Sup. Table 3), and each sub-group contains between 402 and 858 amiRNAs (Fig. 2E, Sup. Table 3). The network relationship of the amiRNAs targeting genes in the library highlights the robustness and coverage of the library design (Fig. 2F, G, Fig. S8). Each amiRNA (pink triangle) targets at least two genes (blue ellipse), and each gene can be targeted by more than one amiRNA (Fig. 2F, G). Thus, our library allows comprehensive targeting of transporter genes and can be useful for studying transporter genes in various contexts.

### Constructing the cell type-specific mTACT toolbox

**Fig 3:**
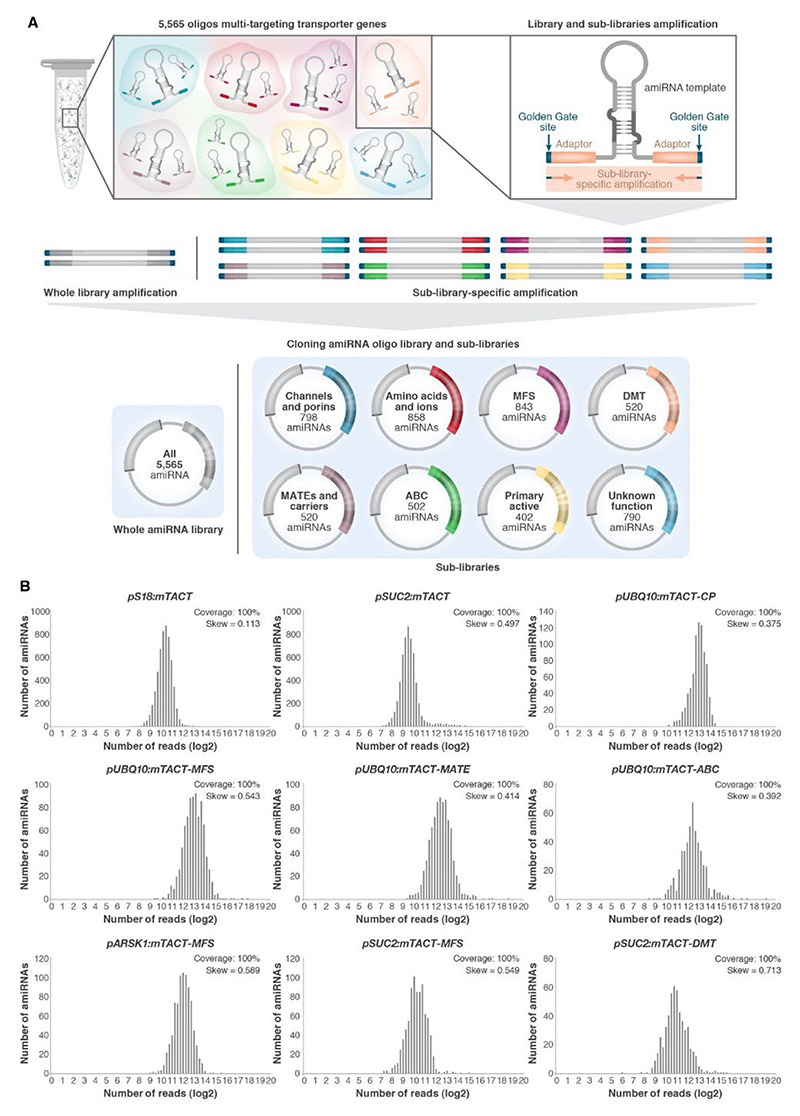
Construction and validation of mTACT sub-libraries. **A)** Illustration of syntheses of 5,565 unique amiRNAs, each targeting two to eight genes from the same transporter gene family. amiRNA library and sub-library amplification are performed using unique adaptors designed to match amiRNAs from a particular transporter family. Numbers of amiRNAs in each sub-library are indicated. **B)** amiRNA distributions for the amiRNA library and sub-libraries based on deep sequencing results. *pS18:mTACT* and *pSUC2:mTACT* were used for the expression of the entire library (5,565 amiRNAs) driven by *S18* and *SUC2* promoters, respectively. *pUBQ10:mTACT-CP, pUBQ10:mTACT-MFS, pUBQ10:mTACT-MATE*, and *pUBQ10:mTACT-ABC* were used for ubiquitous expression of the sub-libraries CP, MFS, MATE, and ABC, respectively; *pARSK1:mTACT-MFS* was used for root expression of the MFS sub-library and *pSUC2:mTACT-MFS* and *pSUC2:mTACT-DMT* were used for phloem companion cells-specific expression of the MFS and DMT sub-libraries, respectively. Coverage indicates the percentage of amiRNAs detected relative to the total number of amiRNAs theoretically synthesized. Skew indicates equal distribution demonstration.

To construct the mTACT libraries driven by cell type-specific promoters, we first generated destination vectors containing cell type-specific promoters with open Golden Gate restriction sites for the amiRNA library cloning (Fig. S1). The transportome-scale forward-genetic libraries transformed into plants would allow the community to reveal phenotypic changes resulting from local and distinct tissue transporter family-members silencing (Fig. S1). To test the efficiency and spatial restriction of amiRNAs, we expressed a GFP-targeting amiRNA under the shoot-specific *SIG6* promoter in a *p35S:GFP* background. Fluorescence imaging revealed shoot-specific knockdown of GFP, with no detectable reduction in other tissues, demonstrating that tissue-specific amiRNA-mediated knockdown is effective and spatially restricted (Fig. S9). Next, we cloned the entire mTACT library (5,565 amiRNAs) under the control of two different cell type-specific promoters *S18* (Marquès-Bueno et al., 2016) and *SUC2* (Truernit and Sauer, 1995) (xylem and phloem companion cells respectively). In addition, using the unique adaptors we designed for each sub-library, we cloned four representative sub-libraries under the constitutive *UBQ10* promoter (Grefen et al., 2010), two representative sub-libraries under the control of the promoter *SUC2*, and one representative sub-library under the root-specific promoter *ARSK1* (Hwang and Goodman, 1995) (Fig. 3A). To confirm that there is a complete and equal representation of amiRNA distributions and that each mTACT library or sub-library contains expected amiRNAs, we deep-sequenced the cloned libraries. The deep sequencing results showed 100% coverage for all libraries, and a bell-shaped representation of the amiRNAs in the library (Skew ranges between 0.113 to 0.713) (Fig. 3B). These amiRNA libraries provide a comprehensive tool for gene silencing and offer a flexible resource for studying gene functions.

**Fig. 4:**
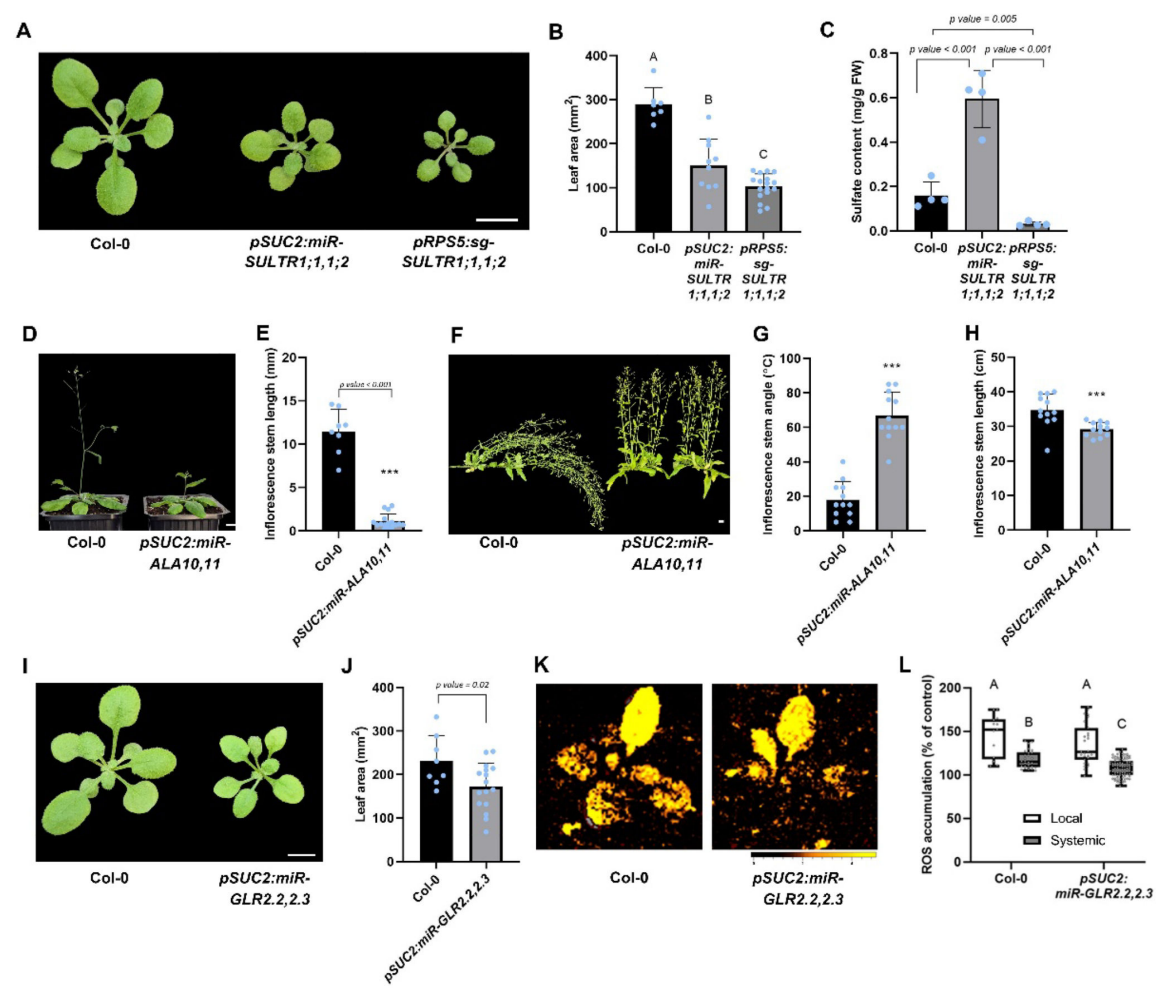
*pSUC2:mTACT* reveals known and novel phenotypes. **A, B)** Leaf area phenotypes **(A)** and quantification **(B)** of 24-day-old Col-0, *pSUC2:miR-SULTR1;1*,*1;2* and *pRPS5:sg-SULTR1;1*,*1;2* plants. Scale bar = 1 cm. Significance was determined using Tukey’s ad-hoc statistical test (treatments marked with different letters are significantly different). **C)** Total sulfate content (mg/g fresh weight) was measured in 45-day-old *pSUC2:miR-SULTR1;1*,*1;2* and respective controls rosette leaves. Significance was evaluated by Student’s t-test, n = 4. **D, E)** Inflorescence stem phenotypes **(D)** and quantification **(E)** of 35-day-old Col-0 and *pSUC2:miR-ALA10*,*11* plants. Scale bar = 1 cm. Significance was evaluated by Students t-test. **F)** Shown are representative images of 47-day-old *pSUC2:miR-ALA10*,*11* plants and respective WT control. At this age, the Col-0 plants bend towards gravity while *pSUC2:miR-ALA10*,*11* plants remain standing straight. Scale bar = 1 cm. **G)** Inflorescence stem angle (main inflorescence stem) of 47-day-old *pSUC2:miR-ALA10*,*11* plants and Col-0 control. Significance was evaluated by Students t-test. n = 12. **H)** Inflorescence stem length measurements (main stem) for 47-day-old *pSUC2:miR-ALA10*,*11* and Col-0. Significance was evaluated by Students t-test, n = 12. **I, J)** Leaf area phenotypes **(I)** and quantification **(J)** of 24-day-old Col-0 and *pSUC2:miR-GLR2*.*2*,*2*.*3* plants. Scale bar = 1 cm. Significance was evaluated by Students t-test. **K, L)** Local and systemic ROS accumulation phenotypes **(K)** and quantification **(L)** in 30-day-old Col-0 and *pSUC:miR-GLR2*.*2*,*2*.*3*, 30 min after application of 1 mM H_2_O_2_ on a single leaf imaged by DCF fluorescence. All experiments were repeated five times with six plants of each genotype per experiment. ROS accumulation was imaged using H 2 DCFDA, n = 48, significance was determined using Tukey’s ad-hoc statistical test (treatments marked with different letters are significantly different).

As a proof of concept, we transformed the mTACT library, driven by the phloem companion cells-specific promoter *SUC2* (Truernit and Sauer, 1995), into a wild-type *Arabidopsis* (Col-0) background. T_1_ seeds were selected for Basta resistance and were collected individually. 786 T_2_ lines were sown in soil and grown under normal long-day conditions. A phenotypic screen focused on shoot growth, leaf color, size, and morphology, from which we found various candidates with altered shoot growth (Fig. S10). We identified a line with a significant phenotype displaying a smaller shoot area compared to Col-0 (Fig. 4A). The phenotype showed dominant segregation in T_2_ plants, suggesting that it is driven by the amiRNA and not by an off-target loss-of-function mutation. This amiRNA line putatively targets two closely related genes from the SULTR family: AT4G08620 (*SULTR1;1*) and AT1G78000 (*SULTR1;2*) (Fig. 4A, B, Sup. Table 5). Hereafter, we refer to this line as *pSUC2:miR-SULTR1;1*,*1;2. SULTR1;1* and *SULTR1;2* encode sulfate transporters that facilitate sulfate uptake from the soil (Rouached et al., 2008). While single mutants were reported to show no noticeable phenotype (Barberon et al., 2008; Rouached et al., 2008), both the *pSUC2:miR-SULTR1;1*,*1;2* and *pRPS5:sg-SULTR1;1*,*1;2* double knockdown or knockout lines exhibit significant shoot growth inhibition, suggesting these transporters have overlapping roles as previously published (Barberon et al., 2008). The stronger phenotype observed in the *pRPS5:sg-SULTR1;1*,*1;2* knockout line compared to the *pSUC2:miR-SULTR1;1*,*1;2* double knockdown line implies that these transporters function in additional cell types or that loss of function in certain cells is not sufficient to fully disrupt their activity. Furthermore, we quantified sulfate levels in the two double knockdown or knockout lines. The vascular-specific knockdown line, *pSUC2:miR-SULTR1;1*,*1;2*, showed a significant increase in total leaf sulfate content compared to both wild-type plants and the ubiquitous knockout line. In contrast, the *pRPS5:sg-SULTR1;1*,*1;2* knockout line exhibited a significant reduction in sulfate levels (Fig. 4C). These opposing phenotypes suggest that while complete disruption of *SULTR* transporters likely impairs sulfate uptake from the soil, restricting their activity specifically to the phloem may block redistribution to sink tissues, resulting in sulfate accumulation in the leaves.

Another phenotype we revealed in the screen is a line showing shorter inflorescence stems compared to the wild type (Fig. 4D). This amiRNA line putatively targets two closely related genes from the ALA family: *AT3G25610* (*ALA10*) and *AT1G13210* (*ALA11*), coined *pSUC2:miR-ALA10*,*11* (Fig. 4D, E, Sup. Table 5). The *Arabidopsis ALA* gene family encodes P4-type ATPases (ALAs), which transport phospholipids across membranes and contribute to lipid homeostasis and membrane dynamics (Poulsen et al., 2015; Botella et al., 2016). ALA10 and ALA11, both cluster-2 members, localize to the ER and exhibit overlapping substrate specificity, including the transport of lysophosphatidylcholine (Davis et al., 2024). ALA10 interacts with ALIS1/5 and the E3 ubiquitin ligase PUB11, which regulates its localization and stability (Salvaing et al., 2020). Despite their biochemical roles, *ala10* and *ala11* single mutants lack distinct phenotypes. However, the *ala10/11* double mutant shows mild reductions in rosette size (Davis et al., 2024), consistent with the delayed growth in *pSUC2:miR-ALA10*,*11* lines generated here (Fig. 4D, E). In addition, compared to wild-type plants, *pSUC2:miR-ALA10*,*11* plants developed slightly shorter inflorescence stems that remained upright at maturity, lacking the typical gravitational bending phenotype (Fig. 4F-H). The reduced elongation and upright posture of *pSUC2:miR-ALA10*,*11* inflorescences may result from disrupted signaling in pathways that regulate stem growth, tissue rigidity or fibers. As ALA proteins influence membrane dynamics, their absence may impair hormone transport or mechanical tissue development essential for vertical support. Growth defects in *ala* mutants were shown to be related to auxin transport due to mislocalization of PIN proteins, along with salicylic acid–dependent growth inhibition (Davis et al., 2024). This points to ALA flippases as key regulators of hormone signaling and membrane protein trafficking essential for proper stem development. Interestingly, a quintuple knockout (*ala8/9/10/11/12*) plant exhibits smaller rosettes and chlorotic leaf lesions linked to salicylic acid (SA)-dependent autoimmunity, which is reversed by expressing an SA-degrading enzyme(Davis et al., 2024), further demonstrating the robust redundancy among cluster-2 ALAs (Gomès et al., 2000; Davis et al., 2024).

Several phenotypes observed in the screen resulted from knocking down genes for which loss-of-function mutant lines had not been previously reported. For example, we isolated a line showing a smaller leaf area compared to wild type (Fig. 4I). This amiRNA line putatively targets two closely related genes from the GLR family: AT2G24720 (*GLR2*.*2*) and AT2G24710 (*GLR2*.*3*), termed *pSUC2:miR-GLR2*.*2*,*2*.*3* (Fig. 4I, J, Sup. Table 5). In addition, systemic ROS accumulation was reported in other genes from the GLR family. The double-mutant *glr3*.*3 glr3*.*6* is blocked in the propagation of systemic electric signals in response to wounding (Mousavi et al., 2013; Toyota et al., 2018; Fichman et al., 2021), highlighting the importance of GLR3.3 and GLR3.6 for systemic signaling. Therefore, we tested rapid systemic ROS signaling in response to a local H_2_O_2_ treatment (ROS-inducing-ROS). The *pSUC:miR-GLR2*.*2*,*2*.*3* line showed reduced systemic ROS signaling compared to Col-0 (Fig. 4K, L), similar to the reported phenotype of *glr3*.*3 glr3*.*6* double mutant (Fichman et al., 2021). The significant developmental phenotype observed here was not reported before for *GLR2*.*2, GLR2*.*3* double knockdown, suggesting that the screen uncovered a possible redundant *GLR* phenotype. *Arabidopsis thaliana* has 20 genes within the *GLR* family (Véry and Sentenac, 2002). Although the ligands of these proteins *in planta* are largely unknown, the GLRs have been shown to be involved in various physiological processes in plants, such as cell signaling, metabolism, wound responses, stomatal aperture function, and development (Mousavi et al., 2013; Nguyen et al., 2018; Farmer et al., 2020). The presence of multiple related genes has hindered genetic efforts to explore the functions of GLRs (Nguyen et al., 2018; Mou et al., 2020).

To investigate the expression levels of the targeted genes in the three knockdown lines, we employed quantitative real-time PCR (qPCR). Unfortunately, although we tested numerous sets of qPCR primers, they proved unreliable and were unable to detect expression at satisfactory levels. We therefore opted for RNA sequencing (RNA-seq) assays to comprehensively assess the expression profiles of the targeted genes in the three knockdown lines *pSUC2:miR-SULTR1;1*,*1;2, pSUC2:miR-ALA10*,*11* and *pSUC2:miR-GLR2*.*2*,*2*.*3*. Differential gene expression analysis was performed using DESeq2 to identify genes that were significantly upregulated or downregulated between the control WT and *pSUC2:miR-SULTR1;1*,*1;2, pSUC2:miR-ALA10*,*11* and *pSUC2:miR-GLR2*.*2*,*2*.*3*, respectively. Approximately 200 differentially expressed genes (DEGs) were identified across these three lines (Fig. S11A-C). However, the FPKM values of the direct amiRNA targeted genes (including different transcripts) in each mutant line showed no significant differences compared with WT (Fig. S11D-F). The lack of significant differences may be attributed to inhibition in translation, rather than affecting RNA levels directly. Additionally, the targeted genes may be expressed in tissues other than the phloem, and the reduction in phloem-specific expression might not be sufficient to detect significant differences. Notably, we did not genetically verify the on-target activity of *pSUC2:miR-GLR2*.*2*,*2*.*3* and *pSUC2:miR-ALA10*,*11* by repeating the phenotype using independent lines. Generating double mutants using combined T-DNA lines, CRISPR, or an intendant amiRNA line would allow validation that the phenotype is not driven by an off-target genetic factor.

## Discussion

mTACT is a genetic toolbox of amiRNAs, each designed to optimally target multiple members of a particular gene family encoding transporter proteins. In total, the mTACT toolbox includes 5,565 amiRNAs, targeting 81.7% of the transportome. Expression of the amiRNAs is driven ubiquitously or using cell type-specific promoters, allowing the design of spatial-specific genetic screens. mTACT can be implemented into any forward-genetic screen. For example, one may transform mTACT into the wild-type background and carry out “classic” phenotypic screens. These screens are likely to reveal previously unreported redundant phenotypes as we demonstrated here. In addition, we speculate that mTACT could be used in combination with more complex forward genetics screens by introducing the amiRNA library into a fluorescent reporter background or into a mutant or over-expression phenotype for a suppressor screen.

The specificity of amiRNA expression driven by tissue-specific promoters is critical. Although the mTACT toolbox targets defined transporter gene families, we must consider whether amiRNA expression produces off-target silencing or perturbs endogenous miRNA networks in the relevant tissue and developmental stage. To ensure that observed phenotypes reflect on-target knockdown, we suggest the following controls: 1) On-target validation: replicate phenotypes with independent amiRNAs, CRISPR, and/or T-DNA against the same genes; concordant results support specificity. 2) Expression analyses: perform RNA-seq and qPCR in the targeted tissue to confirm suppression of intended targets and to assess any unintended effects on endogenous miRNAs and the broader transcriptome. 3) Phenotypic rescue: reintroduce amiRNA-insensitive versions of the targeted genes under their native promoters; rescue of the phenotype strongly indicates specific amiRNA-mediated knockdown.

The mTACT toolbox provides several important improvements over classic genetics and the previously described Phantom amiRNA library (Hauser et al., 2013). First, the amiRNA target combinations that are part of mTACT increase the probability of family knockdown phenotypes. The mTACT library includes 5,565 amiRNAs not present in the synthesized Phantom library. Each amiRNA was designed to target several genes from the same family, and numerous amiRNAs target each gene. Such combinations are ideal for overcoming genetic linkage. Second, the bottom-up amiRNA plotting for each node used in library design maximized the likelihood that redundant functions would be revealed. This design was based on the premise that closely related genes with similar protein sequences will share an overlapping function. Therefore, genes from the same family that do not share a close node on the phylogenetic tree are not targeted by the same amiRNA. Third, the entire amiRNA library was divided into eight sub-libraries, targeting specific functional protein classes. These sub-libraries will allow scientists to flexibly design genetic screens and thus enhance the success rates. Fourth, the ability to drive the amiRNA library under a cell type-specific promoter is an essential addition of mTACT. Cell-file-specific expression of the amiRNA library allows particular processes in the plant to be tackled. The tissue-specific system bypasses issues of lethal phenotypes caused by amiRNAs expressed under the control of the *35S* promoter. Finally, the mTACT amiRNA screening strategy features several advantages over the classical forward-genetic approach. The amiRNAs are dominant in their activity, and therefore the phenotypes are visible at the T_1_ generation. In addition, the 21-nucleotide-long amiRNA can be sequenced to identify putative targets without need for laborious map-based cloning.

Using the mTACT library, we uncovered previously masked genetic contributions to plant signaling and development. We identified a line with reduced shoot area caused by the simultaneous knockdown of *SULTR1;1* and *SULTR1;2*. These genes encode sulfate transporters that mediate root sulfate uptake (Takahashi, 2019; Takahashi et al., 2011). Whereas single-gene disruptions show no overt phenotype, the double knockdown produced marked inhibition of shoot growth, indicating overlapping functions. This finding underscores the value of multiplex targeting to unmask redundancy within transporter families. As follow-up, applying exogenous sulfate to distinct tissues and performing time-resolved recovery assays, together with sulfate-content profiling, could clarify how SULTR1;1 and SULTR1;2 redundantly support sulfate movement across space and time. We also report that the knockdown of *GLR2*.*2* and *GLR2*.*3* attenuates systemic ROS signaling, mirroring the reduced signal reported for *glr3*.*3 glr3*.*6* double mutants and expanding the GLR family’s role in long-distance defense signaling (Xue et al., 2022). In addition, we identified a line with shortened inflorescence stems following simultaneous knockdown of *ALA10* and *ALA11*. These genes encode lipid flippases that are essential for membrane dynamics and lipid homeostasis (López-Marqués, 2021). The double knockdown caused delayed growth and reduced stem elongation, indicating overlapping roles for ALA10/ALA11 in stem development. Because this phenotype has not been reported for single mutants, the mTACT screen uncovered a redundancy-masked trait. As a next step, transverse stem sections could assess changes in fiber formation, secondary xylem thickness, and vascular bundle organization. Together, these findings demonstrate the power of mTACT to expose hidden genetic factors required for the translocation of signaling molecules in plants.

Despite its advantages, the mTACT approach has some limitations. Although miRNAs primarily function in a cell-autonomous manner, meaning they do not freely move in the plant over long- or short-distances (Marín-González and Suárez-López, 2012), several reports have shown that specific miRNAs can move and that this movement is required for plant growth and survival (Brosnan and Voinnet, 2011; Marín-González and Suárez-López, 2012; Liu and Chen, 2018; Skopelitis et al., 2018; Maizel et al., 2020). It has been suggested that RNA molecules have the ability to move from their site of synthesis along the plant, which might allow them to function at a distinct target site (Plavskin et al., 2016; Khakhar et al., 2018; Liu and Chen, 2018; Maizel et al., 2020). Such movement might trigger a systemic response throughout the plant via a non-cell-autonomous activity (Vatén et al., 2011). A set of experiments addressing whether miRNA mobility is developmentally regulated has been conducted recently (Skopelitis et al., 2018). We specifically selected *miRNA159a* as a backbone for mTACT since it was not found to be mobile (Marín-González and Suárez-López, 2012). To rule out the possibility of amiRNA movement in a specific context, an amiRNA targeting GFP or GUS expressed under the respective cell type-specific promoter in the genetic background of ubiquitously expressed GUS and GFP reporters could be performed (Skopelitis et al., 2018). A final caveat is that although we used *in silico* prediction in an attempt to avoid off-target effects of the amiRNAs, we do not know whether the detected phenotypes explicitly result from on-target activity. Therefore, just as with any other forward and reverse genetic approach, it is essential to validate the on-target activity by analyses of independent lines (Schwab et al., 2005; Ossowski et al., 2008; Zhang et al., 2018; Hauser et al., 2019; Takahashi et al., 2020). Validation experiments may involve different amiRNAs targeting the same set of genes, CRISPR, or a combination of T-DNA lines. However, the latter might be a problem if the target genes are genetically linked.

In summary, the mTACT toolbox developed here will allow scientists to overcome functional redundancy of plants in a forward-genetic manner and will allow simultaneous and specific targeting of transporter genes from specified families. mTACT provides a unique toolbox for the scientific community that can be used to study the transport mechanisms of various signaling molecules in plants. The mTACT design and pipeline can be implemented at the genome-scale manner to study redundant families, beyond the plant transportome.

## Materials and methods

### Plant material and growth conditions

All *Arabidopsis thaliana* lines used in this work are in Colombia background (Col-0 ecotype, Salk Institute La Jolla, CA, USA). Sterilized seeds were plated on Murashige & Skoog (MS) x 0.5 (Duchefa Biochemic) medium containing 1% sucrose and 0.8% plant agar (Duchefa Biochemic), pH was adjusted to 5.6-5.8 with 1 M KOH. For transgenic plant selection, antibiotics were added to a final concentration of 50 μg/ml kanamycin (Duchefa Biochemic), 100 μg/ml spectinomycin, 150 μg/ml gentamycin (Duchefa Biochemic), and 30 μg/ml hygromycin (Bio-Gold). Plates with seeds were stratified for 48 hours at 4 °C then transferred to growth chambers (Percival CU41L5) at 21 °C, 100-120 µEm^-2^S^-1^ light intensity under long-day conditions (16 h light/8 h dark). For seed production, plant transformation, crossing, and soil pot assays, seeds were sown on wet soil. Plants were grown in growth rooms under long-day conditions at 21 °C. The following constructs were previously described: *pML1:H2B-GFP* (Roeder et al., 2010), *pKST1:GFP* (Kelly et al., 2017), *pSUC2:YFP, pCO2:YFP, pSCR:YFP, pS18:YFP, pUBQ10:YFP* (Marquès-Bueno et al., 2016).

### Seed sterilization

Seeds were sterilized by vapor-phase sterilization (chlorine fumes) for 2.5 hours in Eppendorf tubes in the presence of 100 ml of 11% sodium hypochlorite and 5 ml of 32% hydrochloric acid in a sealed desiccator.

### Bacterial material and growth condition

All bacteria were grown on LB agar media: 20 g of LB and 15 g bacteriological agar (DIFCO) and added to 1 L double distilled water and autoclaved for 20 minutes at 121 °C. Antibiotics were added according to the specific resistances of bacteria at final concentrations of 50 µg/ml kanamycin, 100 µg/ml carbenicillin, 30 µg/ml hygromycin, 100 µg/ml spectinomycin, 25 µg/ml gentamycin, 10 µg/ml tetracycline, and 25 µg/ml rifampicin. Plasmids were multiplied in chemically competent *E. coli* strain DH5α and extracted with a GenElute plasmid mini extraction kit (Sigma-Aldrich) following the manufacturer’s protocol.

### *Agrobacterium* transformation

Electro-competent *Agrobacterium tumefaciens* strain GV3101 was incubated on ice with 100 ng plasmids for 2 minutes, then electroporated in a MicroPulser (BIO-RAD) (2.2 Kv, 5.8 ms). Bacteria were transferred immediately to 1 ml liquid LB and shaken for 2 hours at 28 °C. Subsequently, bacteria were plated on LB agar plates containing the relevant antibiotics for 2 days at 28 °C.

### Plant DNA extraction and PCR

A “crude” DNA extraction method was used to extract plant genomic DNA to be used as a template for the PCR for sequencing and genotyping purposes. A few young leaves from *Arabidopsis thaliana* (about 100 mg) were placed in a 2 ml round-tip Eppendorf tube and frozen in liquid nitrogen. The leaves were crushed using a tissue-lyser to a thin powder and were homogenized with 400 μl DNA extraction buffer (200 mM Tris-HCL, pH 7.5-8.0, 25 mM EDTA, 250 mM NaCl, 0.5% SDS). The tubes were vortexed for 5 seconds and centrifuged for 1 minute at 13,000 rpm in an Eppendorf mini centrifuge. The supernatant was transferred to a new tube and DNA was precipitated with 300 μl isopropanol and incubated for 5 minutes at room temperature, followed by centrifugation at 13,000 rpm for 5 minutes at room temperature. The pellet was washed with 400 µl 70% EtOH and centrifuged for 1 minute at 13,000 rpm at room temperature, and the DNA pellet was dried and resuspended in 100 µl ultra-pure water. DNA amplification for sequencing and cloning was done by PCR in a Sensoquest labcycler using the Taq Ready Mix (HyLabs) following the manufacturer’s protocol.

### Histochemical GUS staining

For histochemical detection of GUS activity, plant tissues were incubated for approximately 16 hours at 37 °C in 100 mM sodium phosphate buffer (pH 7.0) containing 0.1% Triton X-100, 1 mM 5-bromo-4-chloro-3-indolyl-β-D-glucuronic acid cyclohexylammonium salt (Sigma-Aldrich), 2 mM potassium ferricyanide, and 2 mM potassium ferrocyanide. Tissues were immersed in 70% ethanol until transparent. GUS-stained tissues were imaged using a Zeiss Stemi 2000-C stereomicroscope (Jefferson et al., 1987). Images were captured using ZEN software (Zeiss).

### Cross-sections

Leaf, hypocotyl and petiole cross-sections shown in Fig. 1A and Fig. S2 and S3 were performed by embedding fresh plant material of the indicated ages in 2% low melting temperature agarose (Promega). Freehand sections were then imaged using a Zeiss LSM 710 laser scanning confocal microscope as previously described (Procko et al., 2022).

### Plant phenotyping

For leaf area measurements, plants were grown on soil, one plant per pot, in a growth chamber. A Nikon D5300 digital camera with a Nikon 60 mm Macro Lens was used to photograph plants. The surface area was measured using ImageJ software (http://rsbweb.nih.gov/ij/index.html). Petiole length measurements were performed on 28-day-old plants using a ruler. Petiole angles were quantified using ImageJ software (http://rsbweb.nih.gov/ij/index.html), measurements were performed on 30-day-old plants.

### Confocal imaging

Seedlings were stained in 10 mg/L propidium iodide for 1 minute, rinsed, and mounted in water. Seedlings were imaged on a Zeiss LSM 780 laser scanning confocal microscope with the laser set at 488 nm for GFP, 514 nm for YFP and propidium iodide excitation. Emission filters used were 517–570 nm. Image analysis and signal quantification were done using ZEN lite 2012 software.

### Tissue-specific promoters cloning

Tissue-specific promoters were amplified from genomic DNA or from plasmids containing the specific promoters using Phusion high fidelity Taq polymerase (New England Biolabs), following the manufacturer’s protocol. The amplification was carried out using primers containing restriction sites (Sup. Table 2) necessary for cloning into the destination plasmids pGREEN (pG0229-T) and pBIN PLUS (Hellens et al., 2000; Footitt et al., 2007).

### Library cloning

The promoters used in this study for library construction were previously described and characterized: *pPGP4* (Cho et al., 2007), *pARSK1* (Hwang and Goodman, 1995), *pSIG6* (Hu et al., 2015), *pKST1* (Kelly et al., 2017) were amplified from genomic *DNA; pSUC2* (Siligato et al., 2016), *pCO2* (Siligato et al., 2016), *pS18* (Marquès-Bueno et al., 2016), *pCORI3* (Procko et al., 2022), *pIQD22* (Procko et al., 2022), *pML1* (Savaldi-Goldstein et al., 2007), *pSCR* (Michniewicz et al., 2015) *and pUBQ10* (Grefen et al., 2010) were amplified from plasmids.

A plasmid library expressing a pool of amiRNAs was designed and synthesized as described in Zhang *et al*. (Zhang et al., 2018). The amiRNA library was cloned into pGREEN or pBIN PLUS containing one of the cell type-specific promoters (*SIG6, ARSK1, PGP4, CO2, KST1, SCR, S18, CORI3, IQD22, SUC2, ML1*, or *UBQ10*) using the Golden Gate (Grefen et al., 2010) method. An aliquot of 1 µl of Golden Gate products was transformed into 50 µl *E. coli* DH5α competent cells by heat shock reaction at 42 °C for 30-60 seconds. For each construct, we did 18 transformations. After 1 hour of shaking in 37 °C, samples from three tubes were pooled, and the bacteria were plated on 145/20 mm LB plates containing 50 mg/ml kanamycin resulting in six plates for each construct. Cells were grown overnight in a 37 °C incubator. Bacteria from each plate were scraped into 1 L of sterile LB media with kanamycin, and cultures shaken at 37 °C overnight. For plasmid extraction, we used The QIAprep Spin Maxiprep (Qiagen 20-12162). Next, aliquots of 1 µl of DNA were transformed into the *Agrobacterium pSoup* bacteria system (Hellens et al., 2000) by electroporation. For each promoter, 18 transformations were performed. After 2 hours of shaking at 30 °C, the *Agrobacterium pSoup* were plated on 145/20 mm LB plates containing 50 µg/ml kanamycin, 10 µg/ml tetracycline, 25 µg/ml gentamycin, and 25 µg/ml rifampicin. Three tubes were pooled, resulting in six plates for each construct. Bacteria were grown 2 overnight at 30 °C. Next, each construct was transformed into 6 trays of Col-0 as described above. T_1_ seeds were collected and sown on soil. Beginning when plants were two weeks old, they were sprayed with 0.1% BASTA every 3 days, 3 times in total times.

### Deep sequencing

For deep sequencing analysis of the mTACT transporter library, we started from the *pG0229-T:mTACT* plasmids DNA. By PCR, we created an amplicon for the sense strand of *miRNA159a* using the following primers: forward, gagctttaacttgcccttta; reverse, aagaaaaataaaaaatagagaaggtg. Following amplification, the PCR product was purified using the NucleoSpin Gel and PCR Clean-up system (MACHEREY-NAGEL), and samples were sequenced by Novogene. Deep sequencing data was analyzed using Python. Numbers of reads per amiRNA sequence were determined using the Biopython package. The results were analyzed using Excel.

### amiRNA library construction

Coding sequences for each gene family were obtained from the TAIR 11 database. The nucleotide sequences were then translated to amino acids (protein sequence), and these were used as input to MAFFT (Katoh et al., 2002) to construct a multiple sequence alignment. MAFFT was executed with the algorithm parameter set to “auto” and the maxiterate parameter set to 1000. The resulting alignments were used for maximum likelihood reconstruction of the corresponding gene trees with PhyML (Guindon et al., 2010), using the LG+I+G model and four categories of the gamma distribution. Each internal node in the reconstructed gene trees induces a subfamily of homologous genes, such that subfamilies induced by internal nodes that are further away from the root represent more closely related genes. We then traversed over the internal nodes of the tree and applied the WMD3 program (Ossowski et al., 2008) to obtain a list of potential amiRNA sequences for each subfamily of genes of size *n* ≤ 11. WMD3 potentially outputs a large number of potential amiRNA. To keep only the most efficient amiRNAs, the list of amiRNA was filtered according to the following criteria, such that at most *x* = 8 amiRNAs are selected per internal node:

(1) amiRNAs that target less than two genes with free energy fraction above the threshold value *Ω* were filtered (*Ω* is computed relative to the predicted efficiency of a perfect match). Based on previous studies (Li et al., 2013), *Ω* was set to 0.75.
(2) For each internal node, the amiRNAs were first sorted according to the number of targets with a free energy fraction of at least *Ω*, and then according to the score obtained from the WMD3 output results. Then, in each subtree the amiRNAs were added iteratively from the root to the leaves (in a top-down fashion) until reaching a predefined number of amiRNAs per internal node (here, *x* = 8), or until none were available. An amiRNA was added if either of the two conditions was fulfilled: (a) The sequence of the candidate amiRNA differed by at least *m* = 2 bases from the sequences of all amiRNAs that had been chosen thus far (either in the examined internal node or its ancestral nodes). (b) The sequence of the candidate amiRNA did not fulfill the first condition, but its WMD3 score was better than all other similar amiRNAs.
(3) For internal nodes with less than *x* = 8 selected amiRNAs, an additional procedure was applied to add amiRNAs that target a subgroup of genes in the subfamily (but are not induced by other internal nodes in the tree). To this end, for each gene family, WMD3 was executed on all the target genes with the “must have” parameter set to 2, allowing to design amiRNAs for all possible sets of at least two genes. The candidate amiRNAs were ascribed to the internal node representing the most recent common ancestor (MRCA) of the targeted genes, ranked as in step (2) and added until the total number of amiRNAs per internal node is *x* = 8. However, this option could provide candidate amiRNAs that target genes that are too distant from each other. To this end, we first clustered the targeted genes to monophyletic groups. For each pair of clusters, *A* and *B*, the distance between them was defined as $d(A,B)=\underset{i\in A,j\in B}{\text{min}}\{d(i,j)\}$, where *d*(*i, j*) is the number of internal nodes between genes *i* and *j*. An amiRNA was added if the distance between all clusters was at most 3.
(4) All amiRNAs included in the PHANTOM, L10 library were filtered.
(5) All amiRNAs (sense sequences) and antisense sequences that contained the Bsal sequence or its reverse complement (5’-GGTCTC-3’ and 5’-GAGACC-3’) were removed.

Finally, 5,565 amiRNAs have been designed. Notably, 52.7% of the amiRNAs overlapped with the 2 million amiRNAs from the Phantom database which were not synthesized to date (Hauser et al., 2013).

Next, sense and antisense strands were incorporated into the backbone sequence of *amiRNA159a* (Palatnik et al., 2007), and adaptors were attached according to functional class. For each class, unique forward and reverse primers were designed. The oligonucleotides were synthesized by TWIST Bioscience.

### Network construction

The network graphs were created using Cytoscape (http://www.cytoscape.org/).

### Bioinformatics

DNA sequences alignment was performed using the SnapGene alignment software (http://www.snapgene.com/) and the NCBI BLAST tool (https://blast.ncbi.nlm.nih.gov/Blast.cgi/). Gene sequences and relevant information were obtained from the *Arabidopsis* Information Resource (https://www.arabidopsis.org/).

### Local and systemic ROS imaging

ROS fluorescence for Col-0 and *pSUC:miR-GLR2*.*2*,*2*.*3* plants was imaged using the IVIS Lumina S5 platform (Revvity) as described previously (Fichman et al., 2021). Shortly, plants were fumigated with 50 mM H2DFDA solution for 30 min. A single leaf from each plant was treated with 1 mM H_2_O_2_ and then plants were imaged for 30 min in the IVIS. Data were analyzed using Living Image 4.8.2 software.

### Sulfate Extraction and Quantification from *Arabidopsis* Leaves

For sulfate quantification, rosette leaves of *Arabidopsis* (~100–200 mg fresh weight) were harvested, immediately frozen in liquid nitrogen, and stored at –80 °C until analysis. Frozen samples were ground to a fine powder in a pre-chilled mortar and pestle, transferred into pre-weighed tubes, and the fresh weight was recorded. Sulfate was extracted by adding 1 ml of deionized water (approximately 10 µL per mg FW), followed by vortexing and incubation at 70 °C for 20 minutes. After cooling on ice, samples were centrifuged at 13,000 rpm for 10 minutes at 4 °C, and the resulting supernatant was filtered through a 0.22 μm syringe filter. Sulfate content was analyzed using of 930 Compact IC Flex with an anion-exchange column, and quantified against a standard curve of sodium sulfate.

### RNA-seq

*Arabidopsis thaliana* (Col-0, *pSUC2:miR-SULTR1;1*,*1;2, pSUC2:miR-ALA10*,*11* and *pSUC2:miR-GLR2*.*2*,*2*.*3*) was grown in soil (16 h light / 8 h dark) under normal condition for 15 days. Total RNA was extracted from the leaves and sequenced using the Illumina platform. The raw sequencing reads were processed and aligned to the *Arabidopsis thaliana* TAIR10 using bowtie2. Gene expression levels were quantified using RSEM and normalized using the Fragments Per Kilobase of transcript per Million mapped reads (FPKM) method to account for differences in sequencing depth and gene length. Each line contains three biological replicates. DEGs were identified by |log_2_FC| ⩾ 2 and two-tailed Student’s t-test (P < 0.05).

### Accession Numbers

Sequence data from this article can be found in the GenBank/EMBL data libraries under accession numbers (Supplementary Table 5).

## Supplementary Material

Supplementary Data

## Data Availability

All the data supporting the findings of this study are available within the article and the Supplementary Materials.

## One-sentence summary

An artificial microRNA library targeting redundant families of transport genes in specific cell types exposes hidden regulators that control plant growth and development.
