# Supplementary material for: mTACT: A cell type-specific transportome-scale amiRNA toolbox to overcome functional redundancy in Arabidopsis
Source: Plant Physiol. Author manuscript; Available in PMC 2026 Jan 31. (PMC7618693; doi:10.1093/plphys/kiaf682)
Supplement: Supplementary Data [file EMS211967-supplement-Supplementary_Data.pdf]

Supplemental data

mTACT: A cell type-specific transportome-scale amiRNA toolbox to overcome functional redundancy in Arabidopsis

Moran Anfang[1*], Shir Ben Yaakov[1*], Ning Su[2,3], Anat Shafir[1], Jenia Binenbaum[1], Reem Haj Yahya[1], Xikai Yu[2,3], Carl Procko[4], Hamtual Bar[1], Joanne Chory[4,^], Julian I. Schroeder[5], Yosef Fichman[1], Itay Mayrose[1], and Eilon Shani[1,✉], Yuqin Zhang[2,3✉]

[1] School of Plant Sciences and Food Security, Tel Aviv University, Tel Aviv, 69978, Israel

[2] College of Advanced Agricultural Sciences, University of Chinese Academy of Sciences, Beijing, 100101, China

[3] Institute of Genetics and Developmental Biology, Chinese Academy of Sciences, Beijing, 100101, China

[4] Plant Biology Laboratory, The Salk Institute for Biological Studies, La Jolla, California, 92037, USA

[5] Cell and Developmental Biology Department, School of Biological Sciences, University of California San Diego, La Jolla, CA, 92093-0116 USA

[*] Equal contribution

^ Deceased

✉ Corresponding author

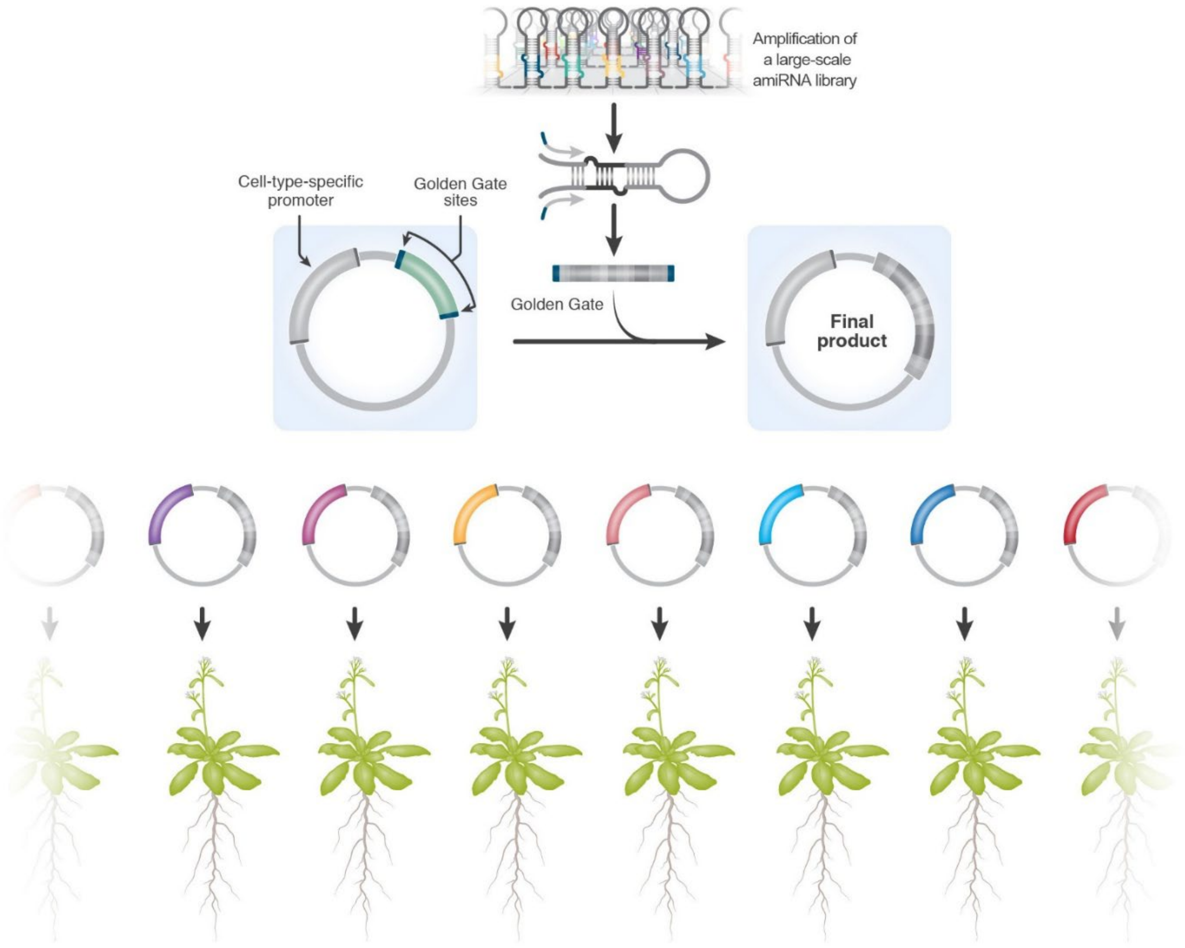

Supplementary Figure S1. Model of cloning and transformation of cell type-specific multi-targeted amiRNA library. Illustration of the cell type-specific amiRNA library cloning and transformation into *Arabidopsis* for genetic screens. The construction steps involve Golden Gate sites and cell type-specific promoter insertion into destination vectors, a large-scale amiRNA library amplification, and ligation into the destination vector using the Golden Gate reaction. After transformation, each plant incorporates a single amiRNA that targets multiple genes from the same family.

Promoter	Full name	Locus ID	Tissue	Expression pattern
ARSK1	*Arabidopsis Root Specific Kinase 1*	*AT2G26290*	Root	*pARSK1:GUS* Cloned for this study
SIG6	*Plastid Transcription Factor*	*AT2G36990*	Shoot	*pSIG6:GUS* Cloned for this study
SUC2	*Sucrose-Proton Symporter 2*	*AT1G22710*	Phloem companion cells	*pSUC2:YFP* (Marquès-Bueno et al., 2016)
PGP4	*Arabidopsis P-Glycoprotein 4*	*AT2G47000*	Root epidermis and cortex	*pPGP4:NLS-YFP* Cloned for this study
CO2	*Lipid-transfer Protein*	*AT1G62500*	Cortex	*pCO2:YFP* (Marquès-Bueno et al., 2016)
KST1	*Voltage-dependent K+ channel*	*X79779*	Guard-cells	*pKST1:GFP* (Kelly et al., 2017)
S18	*MYB Domain Protein 46*	*AT5G12870*	Xylem	*pS18:YFP* (Marquès-Bueno et al., 2016)
SCR	Scarecrow	AT3G54220	Endodermis and bundle sheath	*pSCR:YFP* (Marquès-Bueno et al., 2016)
CORI3	*Coronatine Induced 3*	AT4G23600	Spongy mesophyll	*pCORI3:GUS-mCitrine* (Procko et al., 2022)
IDQ22	*IQ-Domain 22*	AT4G23060	Palisade mesophyll	*pIQD22:GUS-mCitrine* (Procko et al., 2022)
ML1	*Meristem Layer 1*	AT4G21750	Shoot epidermis	*pML1:H2B-GFP* (Roeder et al., 2010)
UBQ10	*Ubiquitin 10*	*AT4G05320*	Constitutive	*pUBQ10:YFP* (Marquès-Bueno et al., 2016)

Supplementary Table 1. Promoters used in this study to drive cell type-specific mTACT. Shown are promoter names, locus IDs, and tissues of expression.

Promoter	Expression	Primers	Length	Resistance in plants
pARSK1	Root	**Fwd**: cacc**aagctt**GAAAATGAATCATTTGTGTTCAGA<br>**Rev:** **gtcgac**TTTCAACTTCTTCTTTTGTGTTAT	1.2 kb	Kana/Basta
pSIG6	Shoot	**Fwd:** cgg**ggtacc**CTCCGAATTCGTCGATTTT<br>**Rev:** ccg**ctcgag**TTCACAGAATCACCAAAGAATAAAAGACTC	373 bp	Kana/Basta
pSUC2	Phloem companion cells	**Fwd**: cag**ggtacc**TACCAGATTTCGGTAAATTGGTAT<br>**Rev**: at**ctcgag**ATTTGACAAACCAAGAAAGTAAGA	2.1 kb	Kana/Basta
pCO2	Cortex	**Fwd:** agg**cgcgcc**TAACTCCATTATTTACGACTGTGCCAC<br>**Rev:** c**ctcgag**AAACTCTTGTTGCATTATTGTCAAATCC	2.1 kb	Kana
pPGP4	Epidermis and cortex	**Fwd:** cacc**ggtacc**GGTAAAGGATTTGGGTCTATTCG<br>**Rev:** ttt**ctcgag**aCGCTCTCTGAAGCCATTAGAGTTT	2.2 kb	Basta
pKST1	Guard cells	**Fwd:** cacc**ggtacc**TCGACTAGAAAATGAAATGAAAAACACC<br>**Rev:** aa**gtcgac**CTGCAGTATTATATATTGCTGCTTC	670 bp	Basta
pS18	Xylem	**Fwd**: cag**ggtacc**TTTTCCTTTGCGTTGACTAA<br>**Rev:** at**ctcgag**ATTTTTGGTTGAGTTAATTG	3 kb	Basta
pSCR	Endodermis/Bundle sheath	**Fwd:** cag**ggtacc**GATTGTGATCCTCTGCAACAA<br>**Rev:** at**ctcga**GGAGATTGAAGGG	2.1 kb	Basta
pCORI3	Spongy mesophyll	**Fwd:** cag**ggtacc**CTCTCTAGAGTTTTCAGAAGATTG<br>**Rev:** ccg**cctcgag**GGACTAACTCTCATTGCTACGA	1.5 kb	Basta
pIQD22	mesophyll	**Fwd:** cag**ggtacc**GGAGGTAAACAGTACATGGTTAAAG<br>**Rev:** ccg**ctcgag**CTAATGAAAGTTACTTGACGAATGAA	3.6 kb	Basta
pML1	Epidermis	**Fwd**: cag**ggtacc**AGTTTCTAAAATGTGCTAAAATTC<br>**Rev:** ccg**ctcgag**CTAACCGGTGGATTCAGGGAG	3.5 kb	Basta
pUBQ10	Constitutive	**Fwd:** cag**ggtacc**CGACGAGTCAGTAATAAACG<br>**Rev:** ccg**ctcgag**CTGTTAATCAGAAAAACTCAG	634 bp	Basta

Supplementary Table 2. List of primers and restriction enzymes used in this study for library construction. Listed are primers and restriction enzymes used in this research for amplifying and cloning the indicated promoters. Lower-case bold letters are nucleotides at restriction enzyme sites, and upper-case letters are promoter sequences.

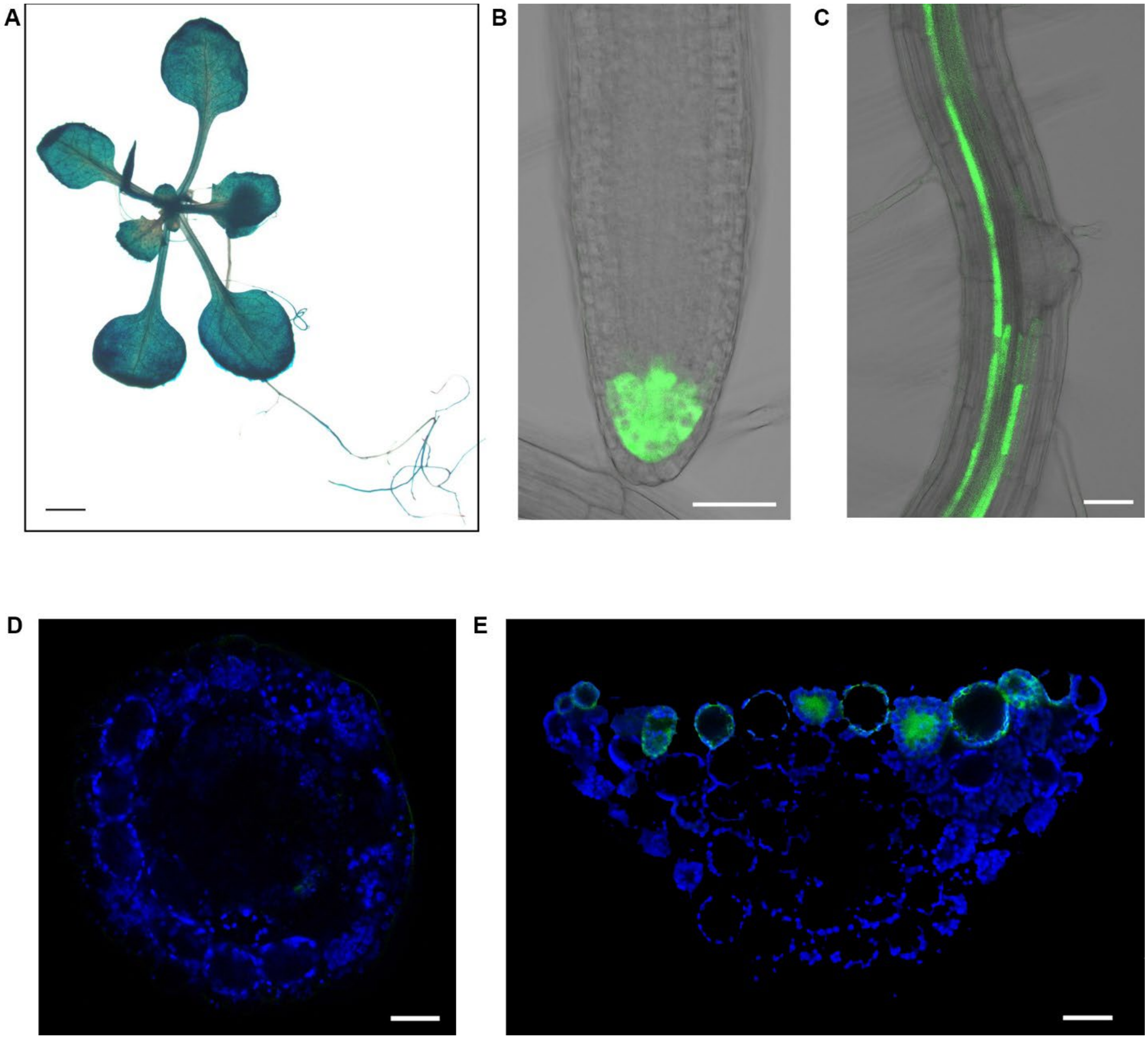

Supplementary Figure S2. *pIQD22* expression pattern. **A)** GUS staining (blue) of 17-day-old *pIQD22:GUS-mCitrine* plant, scale bar = 2 mm. **B-E)** Expression pattern of 17-day-old *pIQD22:GUS-mCitrine* (*mCitrine* shown in green) in the root tip (B), mature root with an emerging lateral root (C), hypocotyl cross section (chlorophyll autofluorescence in blue) (D), and petiole cross-section, from the first pair of true leaves, (chlorophyll autofluorescence in blue) (E), scale bars for images B-E = 50 µm.

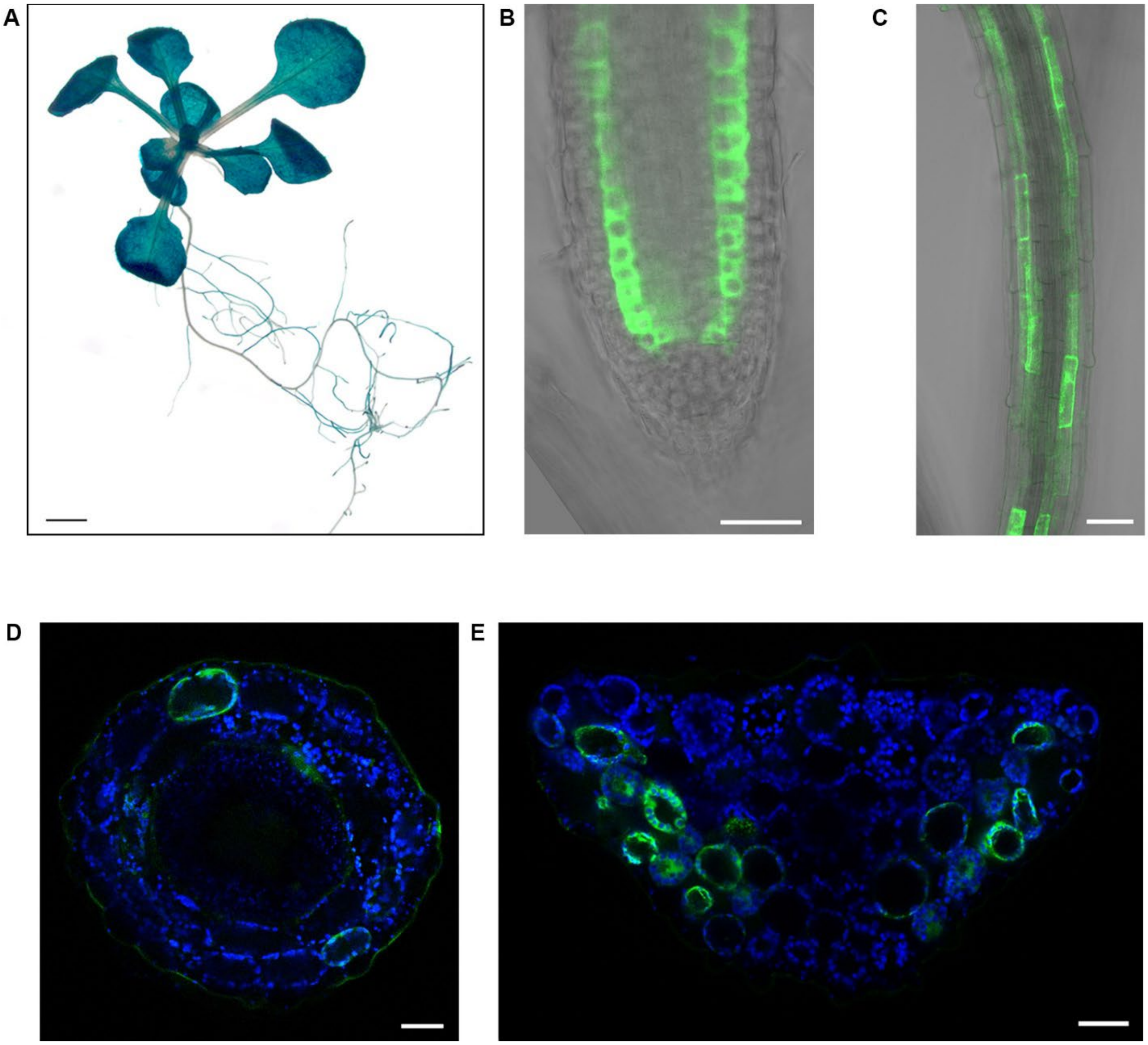

Supplementary Figure S3. ***pCORI3*** **expression pattern**. **A)** GUS staining (blue) of a 17-day-old *pCORI3:GUS-mCitrine* plant, scale bar = 2 mm. **B-E)** Expression pattern of 17-day-old *pCORI3:GUS-mCitrine* (*mCitrine* shown in green) in the root tip (B), the older cortex of the lateral root (C), hypocotyl cross-section (chlorophyll autofluorescence in blue) (D), and petiole section from the first pair of true leaves (chlorophyll autofluorescence in blue) (E). Scale bars for images B-E = 50 µm.

Group	Number of targeted genes	Number of genes in the sub-family	Number of amiRNAs
**mTACT-CP**	164	191	798
**mTACT-APC**	205	261	858
**mTACT-MFS**	181	207	843
**mTACT-DMT**	115	141	520
**mTACT-MATE**	169	224	852
**mTACT -ABC**	97	124	502
**mTACT -PA**	77	89	402
**mTACT -UF**	194	234	790
**ALL**	1,202	1,471	5,565

Supplementary Table 3. Number of targeted genes and number of amiRNAs in each mTACT amiRNA sub-library. Shown are the number of targeted genes, the number of genes in the sub-family, and the number of amiRNAs in each sub-library. CP, channels and porins; APC, amino acid/polyamine/organo-cation, cation carriers, and mitochondrial carriers; MFS, major facilitator superfamily; DMT, drug/metabolite transporter group; MATE, multi-drug and toxic compound extrusion and other carrier transporters; ABC, ATP binding cassette family; PA, primary active transporters; and UF, unknown function.

Class	Primers for amplification	Tm (°C)	Length (bp)
**mTACT-CP**<br>Channels and porins	**Fwd: gaggtctcaattgCATCATCCTGCCTTT**ga	51	300
	**Rev: cggtctcaaaacCCCCTCCATATAACA**gaataa	50	
**mTACT-APC**<br>APC + cation carriers + MC	**Fwd: gaggtctcgattgATCGTTACCCTGCCA**	50	300
	**Rev: cggtctcaaaacGTGGTATGGCTGGTT**g	51	
**mTACT-MFS**<br>MFS	**Fwd: gaggtctccattgTCGTTTAGTGAACCG**ga	50	300
	**Rev: cggtctcaaaacTTGTCCAAACTCATc**gaa	50	
**mTACT-DMT**<br>DMT	**Fwd: gaggtctcgattgGCTACCCGTGATATT**gataga	53	300
	**Rev: gggtctcaaaacGTCATAGCCGAATAG**gaataaa	53	
**mTACT-MATE**<br>Other carriers (MATEs)	**Fwd: gaggtctcgattgTATAGGGAGAGCGGC**gat	54	300
	**Rev: aggtctcaaaacGATTATCCATGGCCG**gaa	54	
**mTACT-ABC**<br>ABC	**Fwd: gaggtctcgattgCGCTTATTGCTATGG**ga	51	300
	**Rev: aggtctcaaaacAAAGTCGTAACAAGG**gaa	51	
**mTACT-PA**<br>Other primary active transpoters.	**Fwd: gaggtctcgattgAGAGTAGGGAACaGC**g	53	300
	**Rev: aggtctcaaaacGCGATTCTGTGACAC**ga	53	
**mTACT-UF**<br>Unknown function	**Fwd**: **gaggtctcgattgCTATGTAGCTGACAC**gatag	52	300
	**Rev: aggtctcaaaacCGACTGTGCCTTCTA**ga	52	
**All**	**Fwd: gaggtctcgattg**gatagatcttgatctgacgatg	52	270
	**Rev: aggtctcaaaac**gaataaataattgttgagtagaaa	48	

Supplementary Table 4. List of adaptors and primers of the mTACT library. Adaptors and primers used for amplification of the mTACT amiRNA library and sub-libraries are listed. Lower-case bold letters are Golden Gate (BsaI) sites, and upper-case bold letters are adaptor sequences.

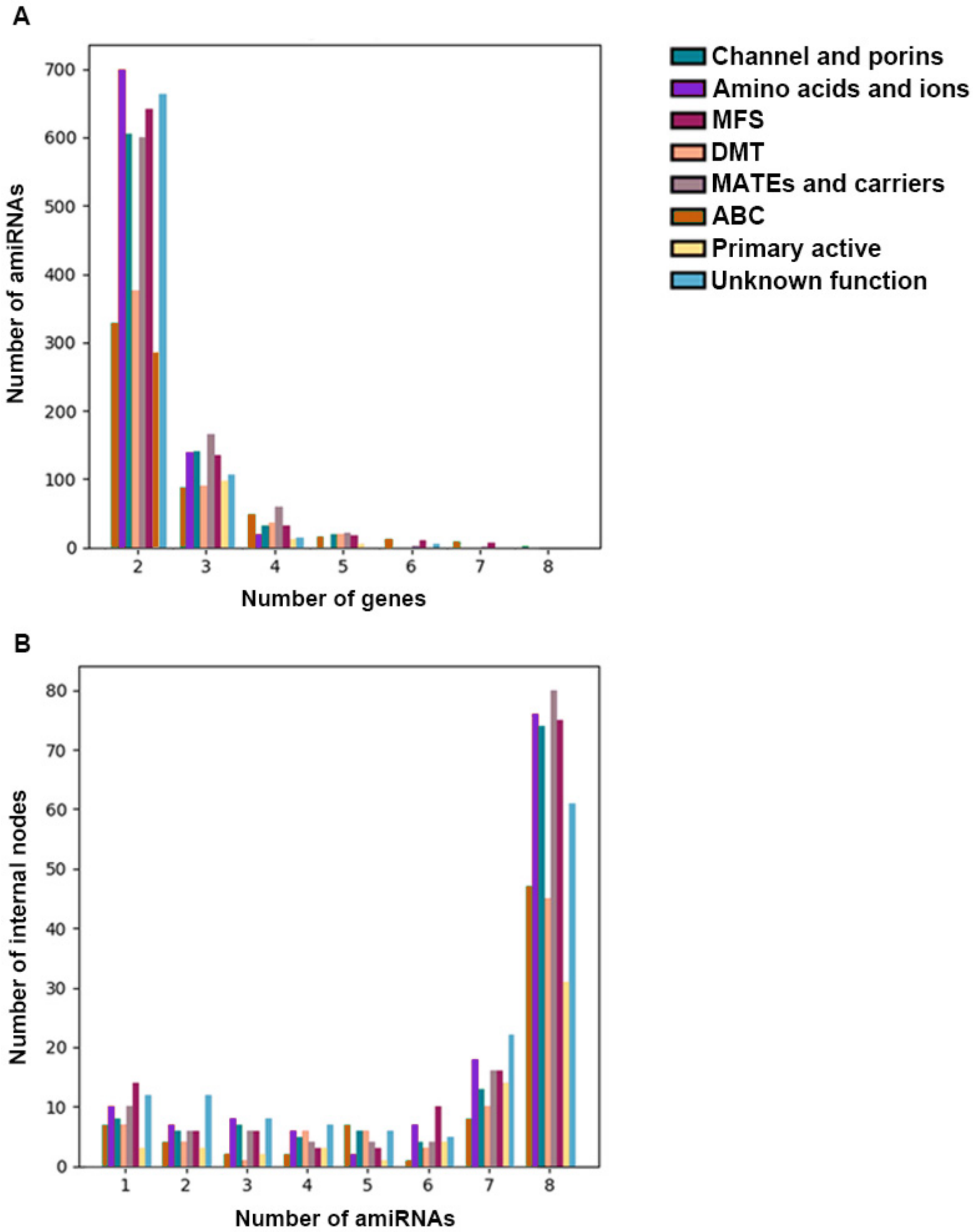

Supplementary Figure S4. Design of the mTACT amiRNA library. A) Number of genes targeted by each amiRNA in each mTACT sub-library. **B**) Number of amiRNAs per internal node in the phylogenetic clade of the gene family.

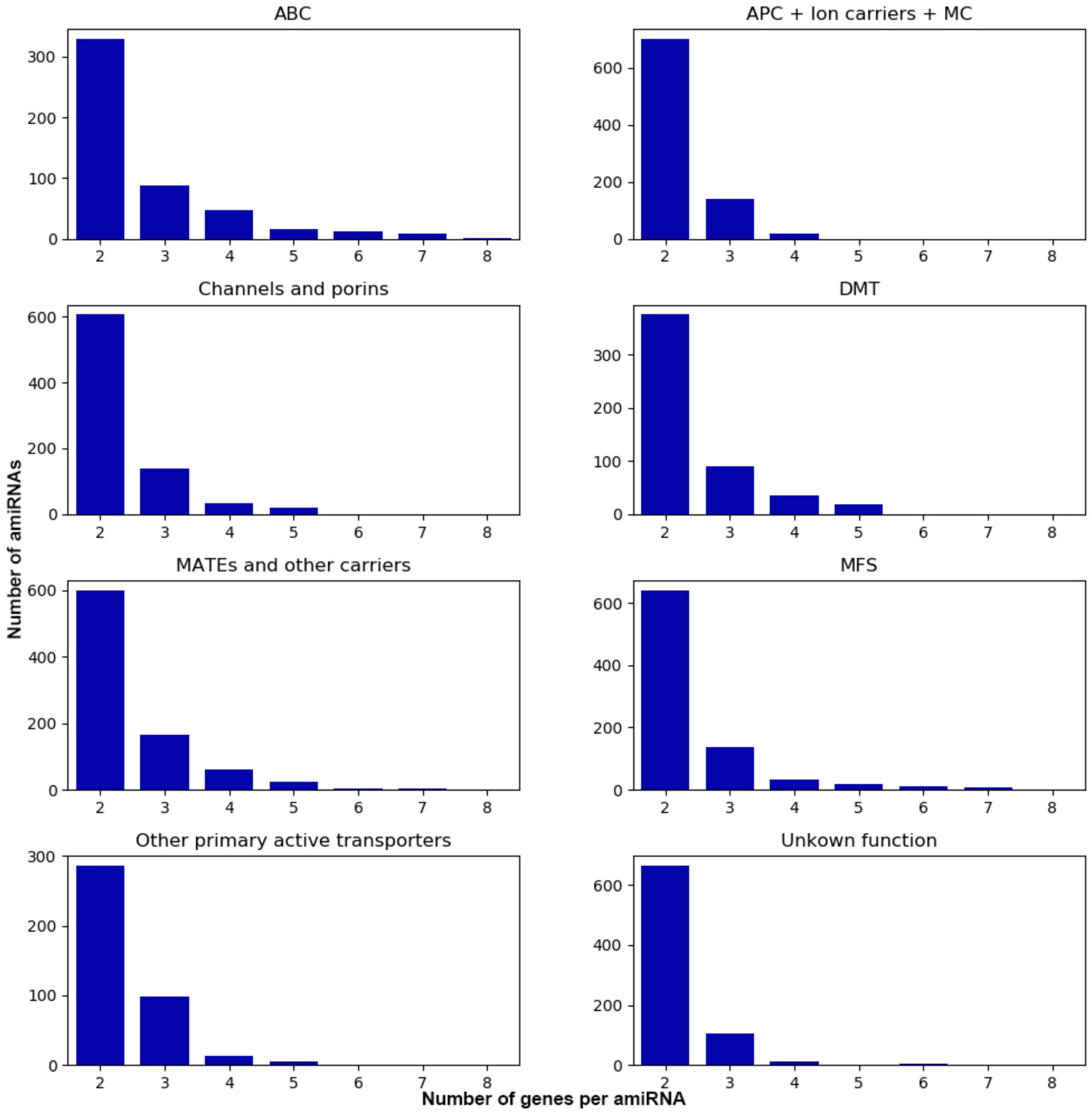

Supplementary Figure S5. Number of genes targeted per amiRNA in each mTACT sub-library. Number of genes targeted by individual amiRNAs in each amiRNA sub-library. The number of genes in an individual internal node was set between two and eight.

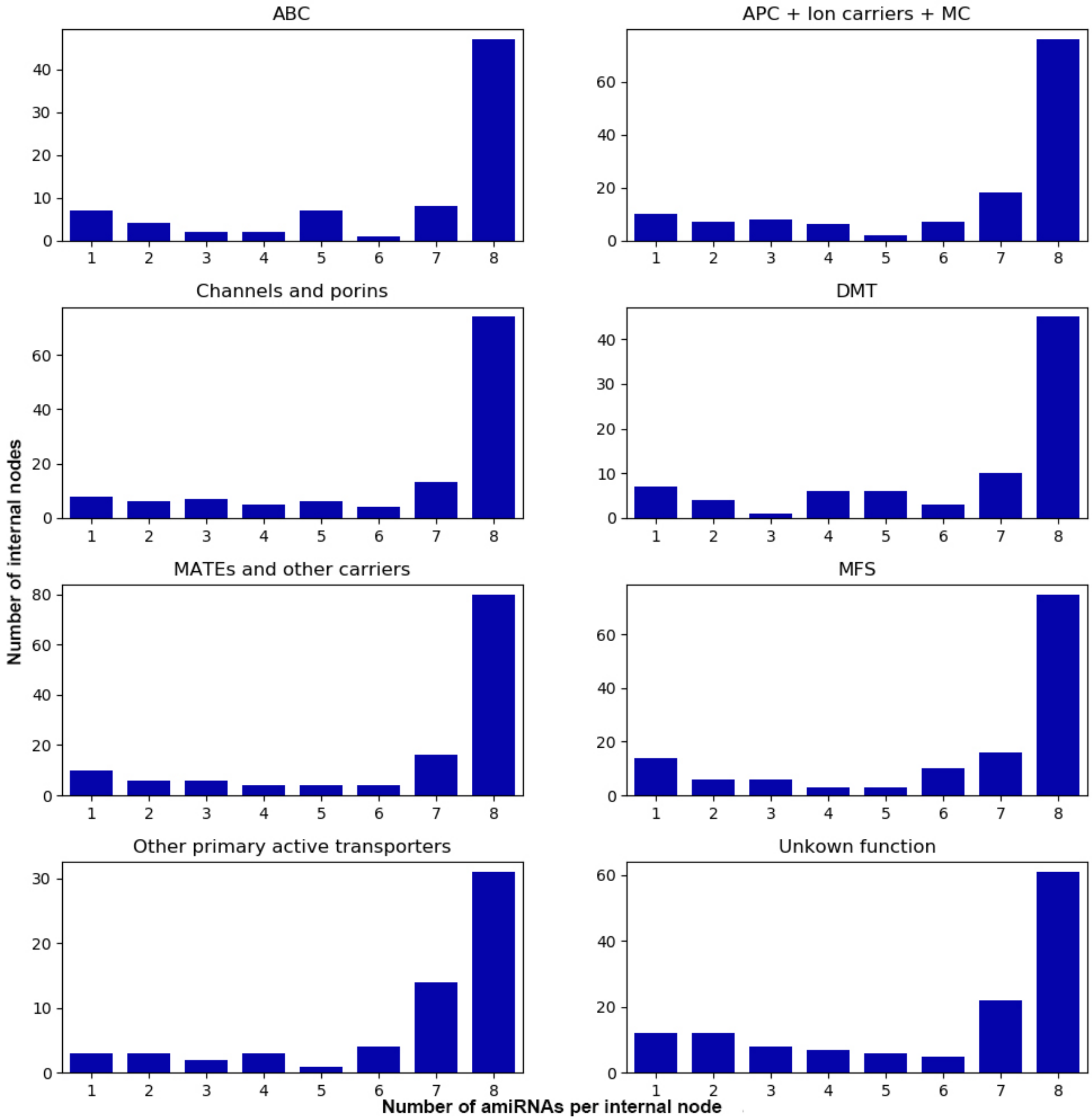

Supplementary Figure S6. Number of amiRNAs per internal node of a gene family. The number of amiRNAs targeting individual internal nodes in each mTACT sub-library. The maximal number was eight, which was chosen in the filtering process.

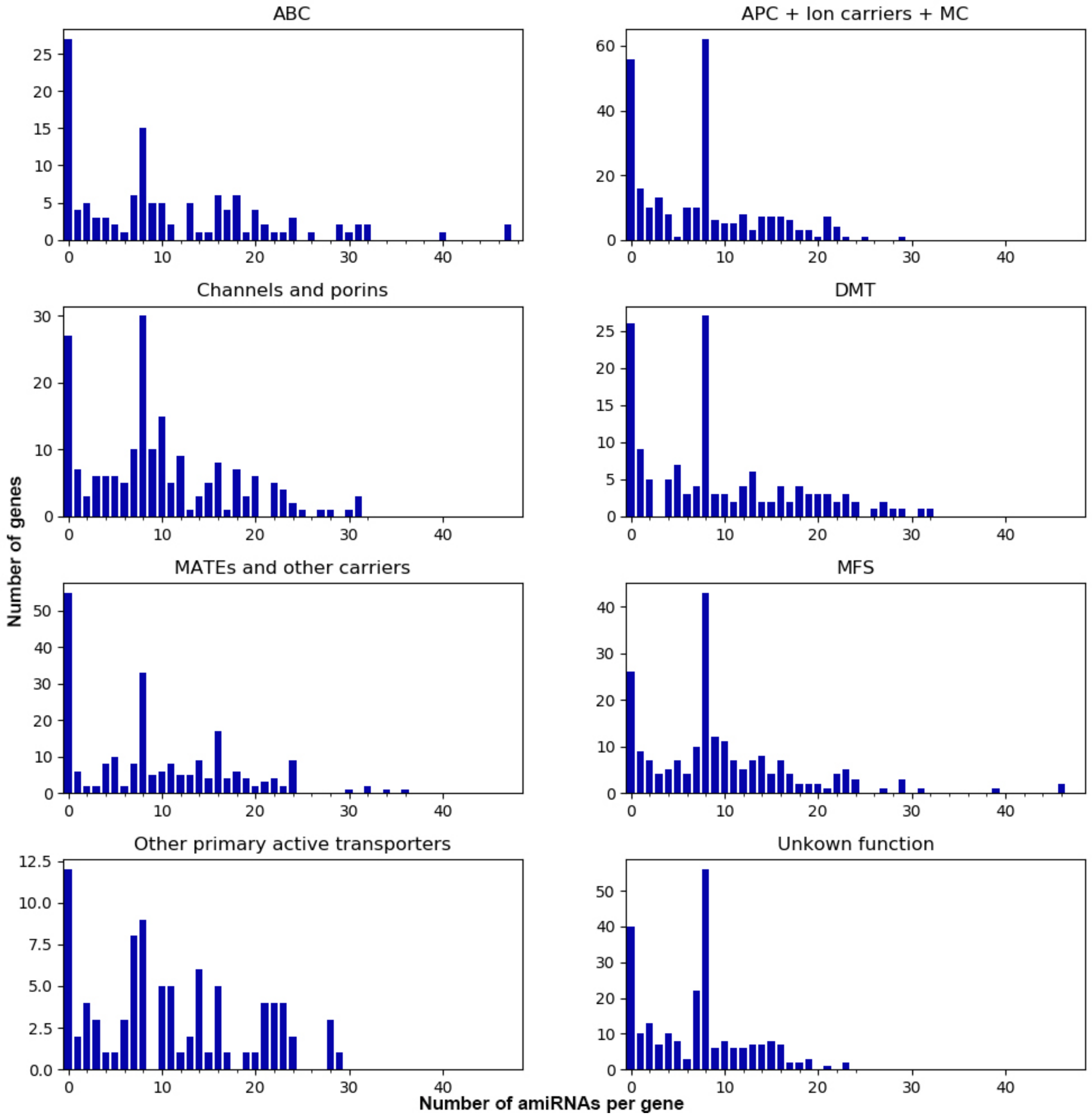

Supplementary Figure S7. Number of amiRNAs per gene. Number of amiRNAs targeting each individual gene. Data are shown for each mTACT amiRNA sub-library. The value zero represents genes that are not targeted.

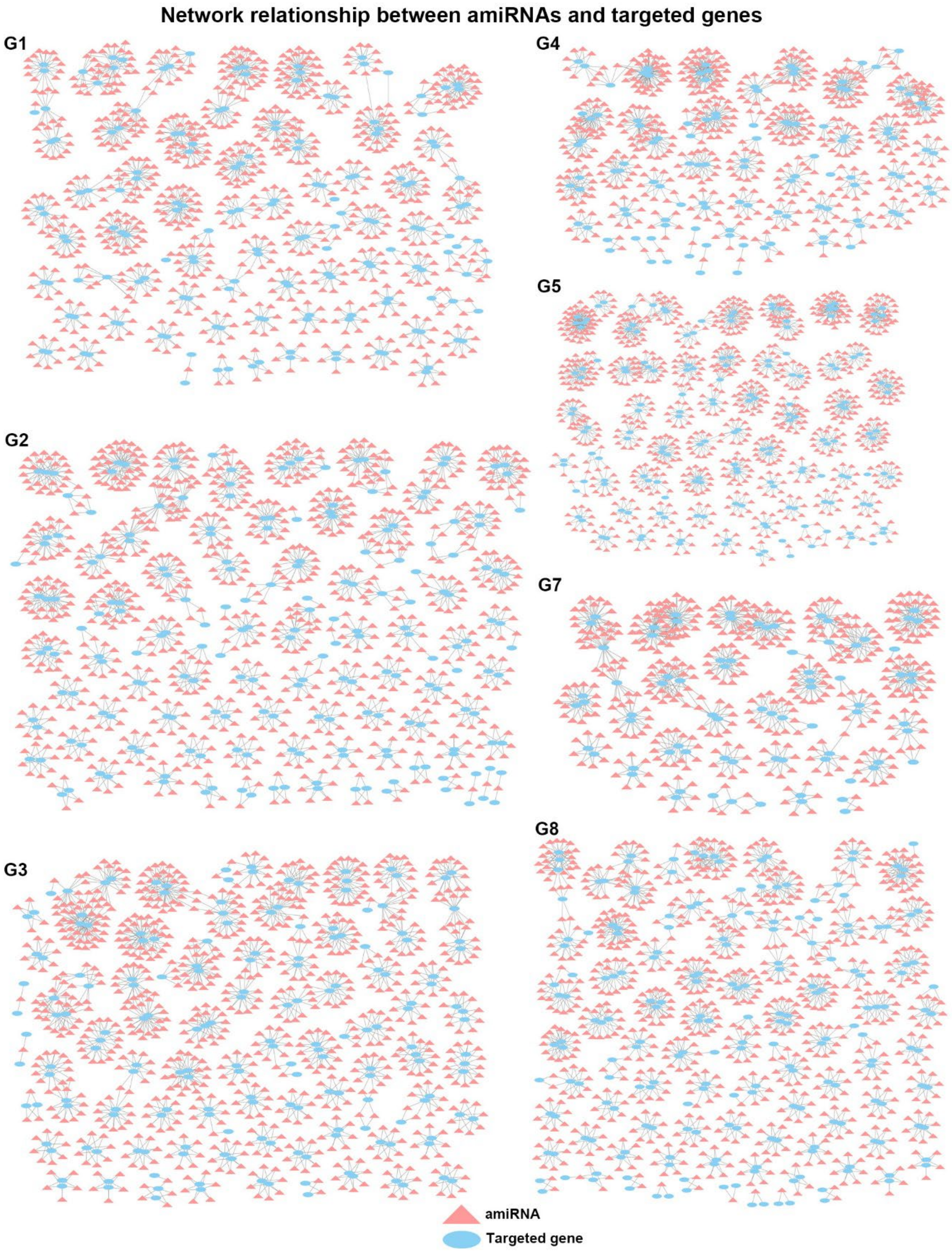

Supplementary Figure S8. Networks describing the relationship between the amiRNAs and targeted genes. Network relationships between amiRNAs (pink triangles) and target genes (blue ellipses) in mTACT sub-libraries. G1: channels and porins (CP); G2: amino acid/polyamine/organo-cation, cation carriers, and

mitochondrial carriers (APC); G3: major facilitator superfamily (MFS); G4: drug/metabolite transporter group (DMT); G5: multi-drug and toxic compound extrusion and other carrier transporters (MATE); G6: ATP binding cassette family (ABC); G7: primary active transporters (PA); G8: unknown function (UF). The ATP-binding cassette (ABC) family network is shown in Fig. 2F.

Line	amiRNA seq	Targeted genes	
pSUC2:miR-SULTR1;1,1;2	TCTATATCCCTAGAACTTCCG	*AT4G08620*	*SULTR1;1*
		AT1G78000	*SULTR1;2*
pSUC2:miR-GLR2.2,2.3	TTTGGGAACTCCAATACGCAC	*AT2G24720*	*GLR2.2*
		AT2G24710	*GLR2.3*
pSUC2:miR-ALA10,11	TAAACCAGTCGTTATACGCTG	*AT3G25610*	*ALA10*
		AT1G13210	*ALA11*

Supplementary Table 5. List of targeted genes in *pSUC2:mTACT* lines. Shown are mTACT lines found in the screen, amiRNA sequences and putative targeted genes.

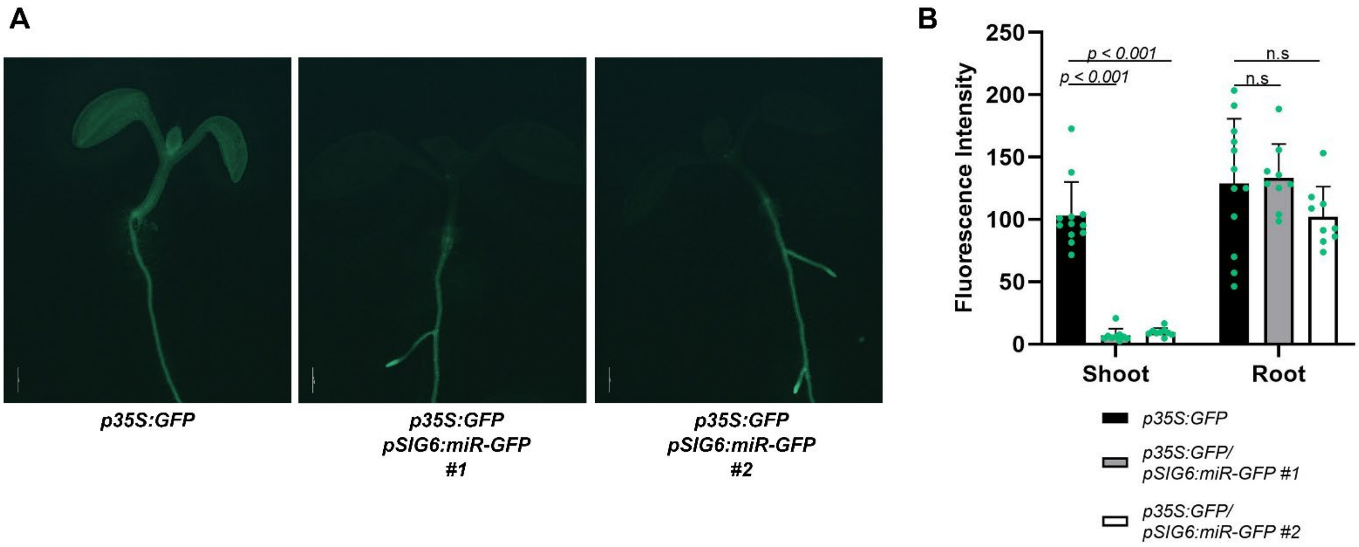

Supplementary Figure S9. Tissue-specific amiRNA knockdown is effective and confined to the promoter-driven expression region. A, B) Shown are representative images **A)** and quantification **B)** of two independent lines of 7-day-old *p35S:GFP/pSIG6:miR-GFP* and respective control. Scale bar = 0.5 mm. Measurements of fluorescence intensity were taken from both root and shoot, n ⩾ 9. Data represent the average +/- SE. Significance was determined using Student's T-test.

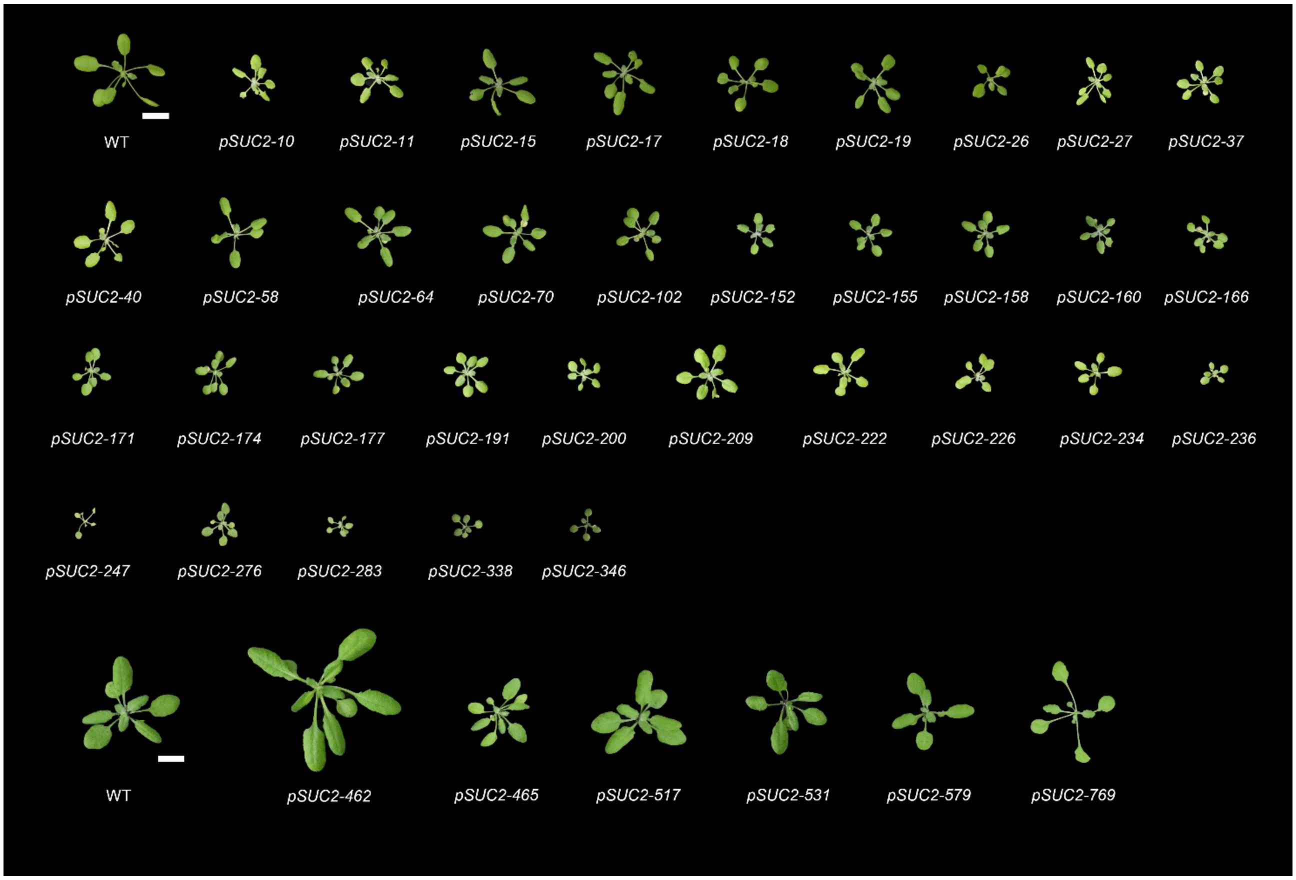

Supplementary Figure S10. Representative candidates from *pSUC2:mTACT* screen for altered shoot phenotypes. Shown are representative images of *pSUC2:mTACT* lines showing altered shoot growth compared to wild-type plants. The plants were grown in soil under normal growth conditions. Images were digitally extracted for comparison. Scale bars = 1 cm.

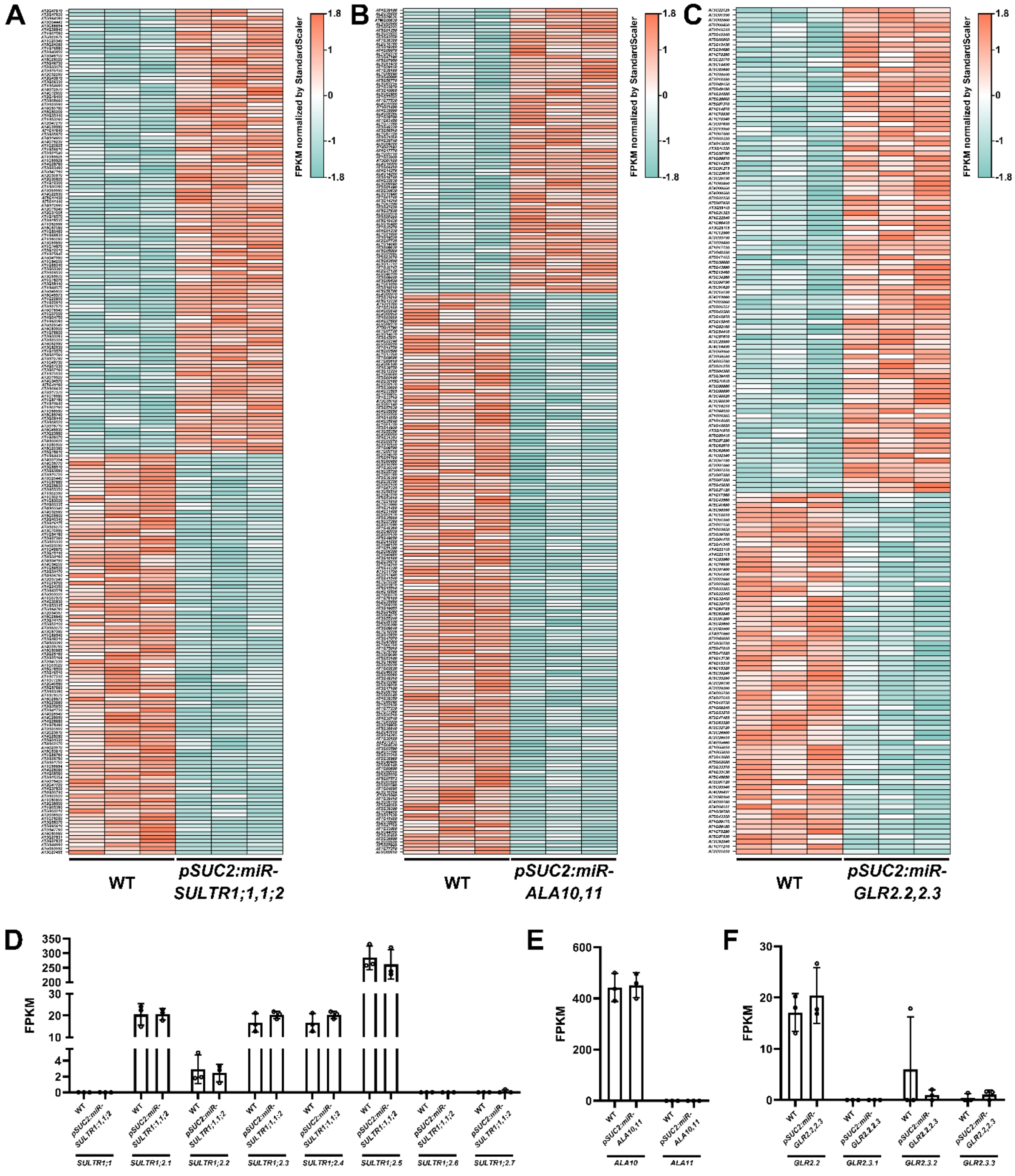

Supplementary Figure S11. The expression level of the targeted genes in the indicated genotypes. A-C) Heatmap of gene expression by RNA-seq for differentially expressed genes (DEGs) in the indicated genotypes *pSUC2:miR-SULTR1;1,1;2* **(A)**, *pSUC2:miR-ALA10,11* **(B)** and *pSUC2:miR-GLR2.2,2.3* **(C)**. Fragments Per Kilobase of transcript per Million mapped reads (FPKM) of each gene in different samples were standardized by StandardScaler. Each line contains three biological replicates. Orange is higher expression and green is lower expression; DEGs defined as genes with a log base two fold change value less than -2 or greater than 2. **D, E, F)** The FPKM of the related targeted genes containing different transcripts in WT and *pSUC2:miR-SULTR1;1,1;2* **(D)**, *pSUC2:miR-ALA10,11* **(E)**, *pSUC2:miR-GLR2.2,2.3* **(F)**.

Each line contains three biological replicates. Among the targeted genes, *SULTR1;2* has 7 transcripts **(D)**, and *GLR2.3* contains 3 transcripts **(F)**, all of which are indicated by "transcript number". Due to the significant differences in the expression levels of different *SULTR1;2* transcripts **(D)**, in order to retain more distinguishable data, the Y-axis was truncated. Data represent the average +/- SE.

References

Kelly G, Lugassi N, Belausov E, Wolf D, Khamaisi B, Brandsma D, Kottapalli J, Fidel L, Ben-Zvi B, Egbaria A, et al (2017) The Solanum tuberosum KST1 partial promoter as a tool for guard cell expression in multiple plant species. J Exp Bot **68**: 2885–2897

Marquès-Bueno MM, Morao AK, Cayrel A, Platre MP, Barberon M, Caillieux E, Colot V, Jaillais Y, Roudier F, Vert G (2016) A versatile Multisite Gateway-compatible promoter and transgenic line collection for cell type-specific functional genomics in Arabidopsis. Plant J **85**: 320–333

Procko C, Lee T, Borsuk A, Bargmann BOR, Dabi T, Nery JR, Estelle M, Baird L, O'Connor C, Brodersen C, et al (2022) Leaf cell-specific and single-cell transcriptional profiling reveals a role for the palisade layer in UV light protection. Plant Cell 1–19

Roeder AHK, Chickarmane V, Cunha A, Obara B, Manjunath BS, Meyerowitz EM (2010) Variability in the control of cell division underlies sepal epidermal patterning in Arabidopsis thaliana. PLoS Biol. 8: 5, e1000367
